# Characterization of Three Novel SINE Families with Unusual Features in *Helicoverpa armigera*

**DOI:** 10.1371/journal.pone.0031355

**Published:** 2012-02-03

**Authors:** Jianjun Wang, Aina Wang, Zhaojun Han, Zan Zhang, Fei Li, Xianchun Li

**Affiliations:** 1 College of Horticulture and Plant Protection, Yangzhou University, Yangzhou, China; 2 Department of Entomology and BIO5 Institute, University of Arizona, Tucson, Arizona, United States of America; 3 College of Plant Protection, Nanjing Agricultural University, Nanjing, China; Texas A&M University, United States of America

## Abstract

Although more than 120 families of short interspersed nuclear elements (SINEs) have been isolated from the eukaryotic genomes, little is known about SINEs in insects. Here, we characterize three novel SINEs from the cotton bollworm, *Helicoverpa armigera*. Two of them, HaSE1 and HaSE2, share similar 5′ -structure including a tRNA-related region immediately followed by conserved central domain. The 3′ -tail of HaSE1 is significantly similar to that of one LINE retrotransposon element, HaRTE1.1, in *H. armigera* genome. The 3′ -region of HaSE2 showed high identity with one *mariner*-like element in *H. armigera*. The third family, termed HaSE3, is a 5S rRNA-derived SINE and shares both body part and 3′-tail with HaSE1, thus may represent the first example of a chimera generated by recombination between 5S rRNA and tRNA-derived SINE in insect species. Further database searches revealed the presence of these SINEs in several other related insect species, but not in the silkworm, *Bombyx mori*, indicating a relatively narrow distribution of these SINEs in Lepidopterans. Apart from above, we found a copy of HaSE2 in the GenBank EST entry for the cotton aphid, *Aphis gossypii*, suggesting the occurrence of horizontal transfer.

## Introduction

Transposable elements (TEs) form a substantial fraction of eukaryotic genomes and are categorized based on their mode of transposition as class-I elements or retrotransposons and class-II elements or DNA transposons [1]. Copy and paste retrotransposons replicate via an RNA intermediate, which is reverse transcribed prior to its reintegration into the genome, whereas cut and paste DNA transposons move through a DNA intermediate. Retrotransposons are the most widespread and enriched class of eukaryotic transposable elements, and can usually be classified into several groups by their replication strategy and structure, including long terminal repeat (LTR) elements, long interspersed nuclear elements (LINEs), and short interspersed nuclear elements (SINEs) [2].

SINEs are nonautonomous repetitive elements with a length of 80–500 bp and depend on LINE elements for their amplification [3]. SINEs can be categorized into families based on sequence similarity and into subfamilies based on the presence of diagnostic nucleotides and/or deletions [3]. Most families are derived from tRNA genes [4], while several are from 7SL RNA [4]–[8] or 5S rRNA [9]–[11]. A typical SINE is composed of three distinct regions: a 5′ terminal RNA-related region (the head) which contains an internal RNA polymerase III (Pol III) promoter, a RNA-unrelated region (the body), and the 3′ tail region that is recognized by the reverse transcriptase (RT) of autonomous partner LINEs during retrotransposition [3].

The majority of SINEs have long been regarded as “selfish DNA parasites or junk DNA”. However, in recent years, striking evidence has accumulated indicating that SINEs are involved in molecular evolution and gene functionality by generating regulatory elements for gene expression, alternative splicing, mRNA polyadenylation, and even functional RNA genes [12]–[15]. For example, more than 5% of the alternatively spliced internal exons in the human genome are derived from Alu SINE [13]. Furthermore, due to the irreversible and random nature of their insertions, SINEs provide excellent molecular markers for phylogenetic analysis via comparison of their insertion sites between different isolates, strains and species [16].

While SINEs are widespread in the animal kingdom in vertebrates and invertebrates, only a few SINEs have been characterized in several insect species, such as Bm1 and BmSE in silkworm, *Bombyx mori* [17]–[18], Lm1 in African migratory locust *Locusta migratoria* [19], Feilai in yellow fever mosquito, *Aedes aegypti* [20], Sine200 in *Anopheles gambiae* [21]–[22], Twin in *Culex pipiens* [23], Talua, Talub, Taluc and Talud in termites [24]–[26], and SINE3-1_TC in *Tribolium castaneum* [27]. Here, we described two tRNA-derived SINE families and one 5S rRNA-derived SINE family in the cotton bollworm, *Helicoverpa armigera*, which is one of the most important pests of cotton in the world. We investigated the genomic structures and insertion regions of these SINEs. The distribution of these SINEs in closely related insect species was also surveyed. Our findings significantly increase the number of known SINEs in insects, and might assist in understanding the roles of retroelements in *H. armigera* genome evolution.

## Materials and Methods

### Insect strains

A laboratory strain of *H. armigera*, generously provided by Dr. Yongan Tang (Institute of Plant Protection, Jiangsu Academy of Agricultural Science, China), was maintained in an insectary kept at 28°C with a photoperiod of 16 h light∶8 h dark on artificial diet. Several third instar larvae randomly picked from the colony were individually flash-frozen in liquid nitrogen and stored in −80°C for subsequent genomic DNA isolation.

### DNA extraction and genome walking

A previous study has shown that transposable elements were enriched within or in close proximity to xenobiotic-metabolizing cytochrome P450 genes in *Helicoverpa zea* [28]. To identity putative novel SINEs in *H. armigera*, we performed genome walking to obtain the 3′-flanking sequence of an insecticide resistance-associated cytochrome P450 gene, *CYP6AE12*, in *H. armigera* [29]. Genomic DNA was isolated from individual third instar larva, using the procedure described by Wang et al [30]. Gene-specific primers (Table S1) based on the known sequence of the cDNA (accession number: DQ256407) and four general primers provided by the Genome Walking Kit (TaKaRa, Dalian, China) were used for every genome walking. PCR products were cloned into pGEM-T Easy vector (Promega, Madison, WI, USA) and sequenced.

### Genomic sequences

While the full genome of *H. armigera* has not been sequenced, a total of 1.963 Mb of genomic DNA sequences covered by 18 BACs was recently available, which approximately represents 0.5% of the *H. armigera* genome [31]. In this work, almost all sequences of HaSE1 and HaSE2 family were retrieved from these BAC sequences downloaded from GenBank (accession numbers: FP340418–FP340435).

### Database search strategy

Database searches were performed and comprise four steps. Firstly, the 3′-flanking sequence of the P450 gene, *CYP6AE12*, was compared with non-redundant databases using the NCBI server with blastn (www.ncbi.nlm.gov/cgibin/BLAST), and sequences of high homology as well as 200 bp upstream and downstream flanking regions were extracted and analyzed for hallmarks of SINEs such as internal RNA polymerase III promoter (Box A and Box B) and target site duplications (TSDs), and the consensus sequence of the first tRNA-derived SINE family in *H. armigera*, HaSE1, was determined. Secondly, the tRNA-related region in the consensus sequence of HaSE1 was searched against non-redundant database using blastn, and the second tRNA-derived SINE family in *H. armigera*, HaSE2, was identified. Thirdly, the unknown 3′ -region of HaSE1 was searched against non-redundant database using blastn, and the 5S rRNA-derived SINE family in *H. armigera*, HaSE3, was identified. Finally, both nucleotide (*nr/nt*) and EST (*est_others*) collections were searched using consensus sequences of these three SINE families as queries to detect new members of SINEs in species other than *H. armigera*.

### Sequence analysis

Sequence alignment was performed using CLUSTAL X [32] with default settings. The A and B boxes of the promoter for RNA polymerase III in HaSE1 and HaSE2 and boxes A, IE, and C in the promoter region of HaSE3 were labeled manually. REPFIND [33] was used to identify identical direct repeats. SINE's tRNA-like structure was checked with tRNAscan-SE [34], using mixed model and cove score cutoff value = 0.01 as default.

In order to test the presence of potential genes in the flanking regions of each SINE insertion, 10 kb sequences in each direction (upstream and downstream) were extracted from the BAC clone sequences and used to search against the non-redundant databases using the NCBI server with BlastX (www.ncbi.nlm.gov/cgibin/BLAST). In the analysis of the GC-content distribution of the elements, 2 kb sequences in each direction were used to calculate GC percent with GEECEE (http://bioweb.pasteur.fr/seqanal/interfaces/geecee.html).

For evolutional analysis, the sequences were aligned using SOAP software [35]. The phylogenetic trees were constructed using the software Mega 5 [36] with the principles of maximum likelihood (ML) and maximum parsimony (MP). To confirm the reliability of ML and MP trees, we also used MrBayes 3.1.2 to conduct a Bayesian inference analysis assuming the optimal models estimated by Modeltest [37]. The reliability of the trees was tested using 1000 bootstrap replications.

### Sequence deposition

The sequences of the HaSE1.1 inserted in the 3′-flanking region of *CYP6AE12* gene and HaSE3 elements obtained by PCR amplification in this study were deposited in GenBank under the accession no. JQ308191–JQ308206.

## Results

### A novel tRNA-derived SINE family, HaSE1, in *Helicoverpa armigera*

A novel tRNA-derived SINE family, designated as HaSE1 (the first SINE family discovered in *H. armigera*), was identified by genome walking and subsequent database searches. The HaSE1.1 element identified in the 3′-flanking sequence of the P450 gene, *CYP6AE12*, is 324 bp in length and located at 649 bp downstream of the translation stop codon of the *CYP6AE12* gene in the reverse orientation. A total of 21 full length sequences with high homology to HaSE1.1 were identified from non-redundant database, and named HaSE1.2-HaSE1.22 (Table 1). Figure S1 shows the alignment of these sequences and the deduced consensus sequence. As shown in Figure S1, these sequences present the typical structural features of the tRNA-derived SINE elements: all HaSE1 copies are flanked by 5 to 18 bp short direct repeats (DRs) or target site duplications (TSDs), presumably generated during retroposition; the A and B boxes of the RNA polymerase III promoter as well as a tRNA-like region were found at the 5′-end; various numbers of perfect or imperfect TGA trinucleotide repeats were found at the 3′ -end.

**Table 1:** Full length HaSE1 and HaSE2 elements identified in this study.

Name	GenBank	Location (nt)^a^	Length (nt)	Identity (%)^b^	GC content (%) 5′/3′
HaSE1.1	JQ308191	1082–759	324	81	ND
HaSE1.2**-1** *	FP340421	20817–21088	272	81	37/32
HaSE1.2**-2** *	FP340425	2038–2309	272	81	37/32
HaSE1.3-1	FP340422	76725–77124	400	95	41/41
HaSE1.3**-2**	FP340436	65754–65354	401	95	35/36
HaSE1.4	FP340423	15381–15782	402	97	33/38
HaSE1.5-1*	FP340434	83754–84151	398	93	40/41
HaSE1.5-2*	FP340425	33395–32998	398	93	40/41
HaSE1.5-3*	FP340421	52178–51781	398	93	40/41
HaSE1.5-4	FP340430	3597–3200	398	93	40/37
HaSE1.6	FP340427	56774–57163	390	96	40/39
HaSE1.7	FP340428	23925–23540	386	96	35/36
HaSE1.8	FP340430	48647–49045	399	96	36/35
HaSE1.9	FP340430	63322–62941	382	94	36/31
HaSE1.10-1	FP340430	10685–10341	345	83	37/37
HaSE1.10-2*	FP340434	76862–77152	291	80	38/35
HaSE1.10-3*	FP340425	40287–39997	291	80	38/35
HaSE1.10-4*	FP340421	59070–58780	291	80	38/35
HaSE1.11-1	FP340430	59655–60021	361	77	34/29
HaSE1.11-2*	FP340421	106276–106636	361	79	35/29
HaSE1.11-3*	FP340425	87493–87853	361	79	35/29
HaSE1.11-4*	FP340434	29656–29296	361	79	35/29
HaSE1.12	FP340432	12205–11822	384	96	40/38
HaSE1.13	FP340432	92706–93093	388	97	39/38
HaSE1.14	FP340437	3418–3033	386	95	34/35
HaSE1.15	FP340438	10122–10510	389	96	34/37
HaSE1.16	FP340438	5597–5977	381	97	36/36
HaSE1.17	FP340432	62457–62840	384	92	34/43
HaSE1.18-1	FP340437	61245–61648	404	86	34/36
HaSE1.18-2	FP340423	62742–63144	403	85	35/41
HaSE1.19	FP340422	92608–92224	385	95	40/38
HaSE1.20	FP340435	100427–100075	353	82	32/38
HaSE1.21	FP340435	71654–72034	381	80	35/42
HaSE1.22	EU920879	68–460	393	96	ND
HaSE2.1	HQ453271	3805–3517	289	96	40/40
HaSE2.2-1*	FP340425	94643–94943	301	97	33/30
HaSE2.2-2*	FP340434	22506–22206	301	97	33/30
HaSE2.3	FP340437	75891–76196	306	94	38/36
HaSE2.4	FP340431	76086–75777	310	96	42/35
HaSE2.5	FP340433	83917–83633	285	97	39/37
HaSE2.6	FP340432	95944–95659	286	93	33/38
HaSE2.7	FP340435	124636–124393	244	66	33/35

a No obvious target site duplications (TSD) were identified for HaSE2.6 and HaSE2.7. Only the locations corresponding to the aligned and conserved SINE sequences were indicated.

b Identity to the corresponding consensus sequence.

*These copies of a specific element showed 100% nucleotide identity to each other.

ND: not determined.

**Figure 1:**
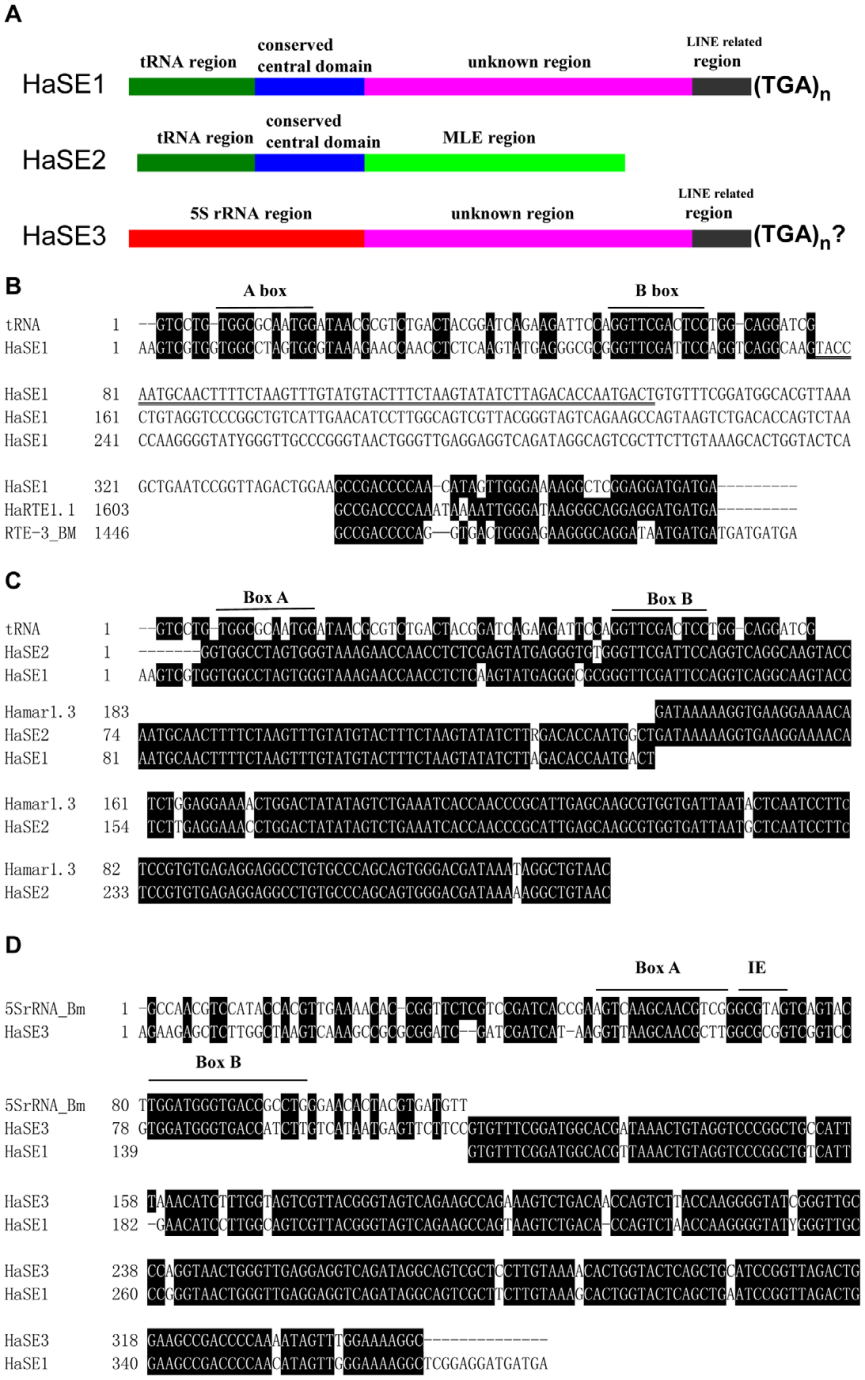
The structure and consensus sequences of HaSE1, HaSE2 and HaSE3. (A) Schematic representation of consensus sequences of HaSE1, HaSE2 and HaSE3. (B) HaSE1 consensus sequence aligned with *Drosophila melanogaster* tRNA^Arg^ sequence (accession number: V00243) and 3′-tail sequences of two LINE elements, HaRTE1.1 and RTE-3 BM. The RTE-3 BM sequence was obtained from Repbase Update database. Nucleotides shaded in black are conserved across sequences. Box A and Box B are the RNA polymerase III promoter sequences. The conserved central domain is underlined. (C) HaSE2 consensus sequence aligned with tRNA-related region and conserved central domain of HaSE1 as well as partial sequence of one *mariner*-like element, *Hamar1.3*, in *Helicoverpa armigera* (Accession number: HM807607). Box A and Box B are the RNA polymerase III promoter sequences. (D) HaSE3 consensus sequence aligned with 5S rRNA from *Bombyx mori* (Accession number: K03316) and 3′ -region of HaSE1. Boxes A, IE and C are the RNA polymerase III promoter sequences.

The consensus sequence of the HaSE1 family members is 386 bp long (Figure 1A and B). It includes a short A-tag followed by a 74-bp tRNA-related region at the 5′-end (58% identity to 72-bp tRNA of *Drosophila melanogaster*, Figure 1B). The tRNA-related head retains the ability to fold into a cloverleaf structure, and the scanning for secondary structures indicated that HaSE1 was derived from a pseudogenic tRNA^Glu^ (Figure S2). The body part is not related to any known sequences. Comparative analysis showed that the match between the HaSE1 elements and the consensus sequence ranged from 77% to 97%, with a median similarity of 89%.

**Figure 2:**
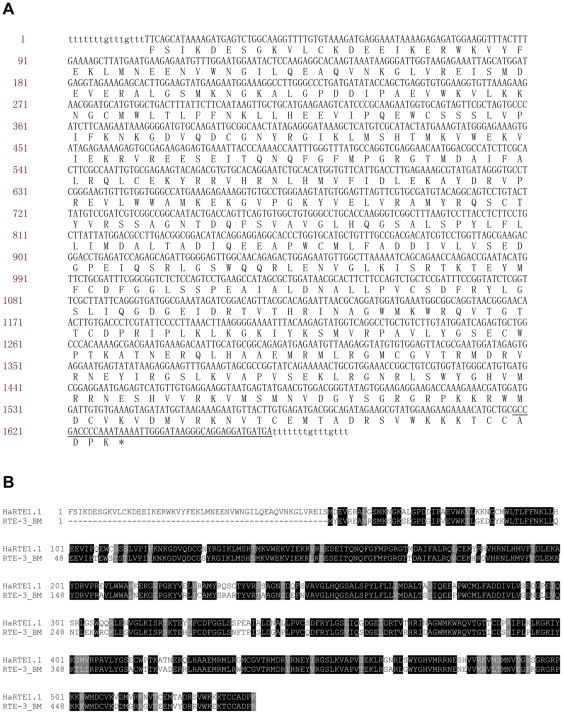
Identification of the partner LINE element for HaSE1 and HaSE3. (A) The nucleotide sequence and conceptual translation of the LINE element HaRTE1.1. The 3′ end sequences similar to HaSE1 and HaSE3 were underlined. Flanking direct repeats are indicated in lowercase. (B) Alignments of RTase amino acid sequences of HaRTE1.1 and RTE-3-BM. Identical amino acids are shown in black boxes and similar amino acids are highlighted in gray boxes.

In a computer assisted similarity search, we found that the 3′-terminal 43 bp fragment of the HaSE1 consensus sequence was very similar (82% identity) to the 3′-end of HaRTE1.1 (Figure 1B), a member of the novel LINE family HaRTE1 we identified in a BAC clone of *H. armigera* (accession number: FP340435; position: 64987–66631). Thus, this region was designated as 3′ -LINE-related region. The HaRTE1.1 element was 1,645 bp long, flanked by 15 bp TSDs, encoded an RTdomain with 42% amino acid sequence identity to that of the RTE-3-BM in *B. mori* [38], and was terminated by a region of TGA trinucleotide repeats in the short 3′ UTR (Figure 2A and B).

In addition, we also identified several 5′-truncated forms of HaSE1 and these elements were named as HaSE1.23-HaSE1.43 (Table 2). The lengths of these truncated HaSE1 sequences varied between 75 bp and 356 bp. These sequences are flanked by 5–17 bp perfect or nearly perfect TSDs (Table 2).

**Table 2:** 5′-truncated HaSE1 elements identified in this study.

Name	GenBank	Location (nt)	Length (nt)	TSD	GC content (%) 5′/3′
HaSE1.23	FP340424	93555–93835	281	atgttatgggcatcgc	38/32
HaSE1.24	FP340424	88889–88588	302	aatcagaag	31/35
HaSE1.25	FP340433	3655–3925	271	agagctgaatcagctg	35/36
HaSE1.26	FP340437	33829–34075	247	taatttt	31/33
HaSE1.27	FP340438	19747–19470	278	atcctcta(g)cagtc	38/35
HaSE1.28	FP340421	1032–1387	356	aaggggtaa	35/35
HaSE1.29-1*	FP340425	747–491	257	atctta	35/38
HaSE1.29-2*	FP340421	19526–19270	257	atctta	35/37
HaSE1.30	FP340421	9640–9754	115	ataaaagtatgtcttg	34/37
HaSE1.31	FP340435	88927–88681	247	atttctc	34/35
HaSE1.32	FP340435	105108–105257	150	tgatga	38/34
HaSE1.33	FP340431	1576–1733	158	tggtttt	38/40
HaSE1.34	FP340421	9640–9754	115	ataaaagtatgtcttg	34/37
HaSE1.35	FP340435	57601–57855	255	aactagtact	37/38
HaSE1.36	FP340435	55804–55957	154	tggaaagcta	38/38
HaSE1.37	FP340435	13183–13068	116	atatttttt	35/33
HaSE1.38	FP340435	111769–111913	145	acagggaag	29/31
HaSE1.39	FP340435	52746–52855	110	gtattttagaccccg	46/38
HaSE1.40	FP340437	30962–31090	129	ttacccagaaatcaa	37/32
HaSE1.41	FP340433	27046–27120	75	tggctgaaaagaaatga	32/34
HaSE1.42	FP340435	21259–21074	186	ttactttagcagct	37/33
HaSE1.43	FP340427	24173–24329	157	gaaaaagcacttaagg	40/47

TSD: target site duplication.

*These copies of a specific element showed 100% nucleotide identity to each other.

### The second tRNA-derived SINE family, HaSE2, in *Helicoverpa armigera*

Using 74-bp tRNA-related region of HaSE1 as a query, our additional search has identified 7 sequences (HaSE2.1–HaSE2.7) of the second tRNA-derived SINE family, HaSE2 (Table 1, Figure S3). The 5′-structure of the 286 bp consensus sequence of HaSE2 is highly similar to that of HaSE1 (94% identity over a 132-nt region), including a 70-bp tRNA-related region immediately followed by a 62 bp conserved central domain (Figure 1A and C). Note that the 154 bp 3′-region showed high identity (98%) with one *mariner*- like element, *Hamar1.3*, in *H. armigera* [39] (Figure 1C). Five out of the 7 HaSE2 elements are flanked by TSDs, but no simple repeat or long stretches of A and T was found in 3′-tail of HaSE2 elements (Figure S3). We found that HaSE2.7 is more degenerate than other SINEs, sharing only 66% sequence identity with the HaSE2 consensus sequence.

### HaSE1 and HaSE2 flanking regions characterization

To reveal the insertion positions of the two tRNA-derived SINE families, we examined the associations of HaSE1 and HaSE2 with putative genes in the *H. armigera* genome. The results showed that a total of 40.9% (9/22) of full length HaSE1, 27.3% (6/22) of 5′-truncated HaSE1 and 42.9% (3/7) of HaSE2 elements inserted within introns (Table S2, Table S3), suggesting the tendency of HaSE1 and HaSE2 to reside within intronic regions. There are no copies of HaSE1 and HaSE2 located in exons. A total of 5 copies of full length HaSE1, 7 copies of 5′-truncated HaSE1 and 2 copies of HaSE2 were found to be located close to other TEs (Table S2, Table S3). One copy of HaSE1 was found in microsatellite DNA loci.

**Figure 3:**
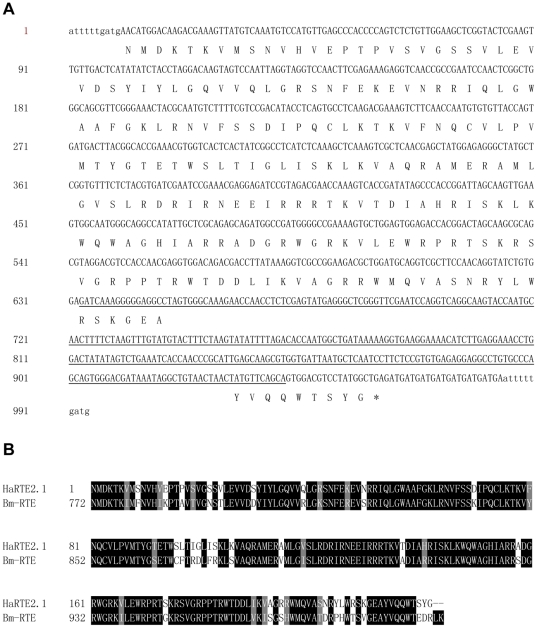
Identification of the HaSE2.4-nesting LINE element HaRTE2.1 in *Helicoverpa armigera*. (A) Nucleotide sequence and conceptual translation of HaRTE2.1. Flanking direct repeats are indicated in lowercase. The inserted HaSE2.4 sequence is underlined. (B) Alignments of RTase amino acid sequences of HaRTE2.1 with Bm-RTE (accession number: ADI61812). Identical amino acids are shown in black boxes and similar amino acids are highlighted in gray boxes.

Interestingly, analysis of the 5′- and 3′-flanking sequence of HaSE2.4 revealed a new LINE element in *H. armigera*, which was named as HaRTE2.1. The inserted HaSE2.4 is in the sense orientation compared to the ORF of HaRTE2.1 (Figure 3A). The HaSE2.4-nesting HaRTE2.1 is 994 bp long in total, flanked by 10 bp TSDs, encoded an RTdomain with 81% amino acid sequence identity to that of the Bm-RTE in *B. mori* (accession number: ADI61812), and was terminated by a region of TGA trinucleotide repeats in the short 3′ UTR (Figure 3A and B).

In addition, we examined the GC contents of the flanking regions of HaSE1 and HaSE2 elements. We found that the insertion sites of these two SINE families had a similar GC content with that of the sequenced part of the *H. armigera* genome (36.1%) [31]. The average GC contents of 5′- and 3′- flanking regions of HaSE1 were 36.5% and 36.4% for full length elements, and all 35.8% for 5′ -truncated elements, and that of HaSE2 were 36.4% and 35.1%, respectively (Table 1, Table 2).

### Distribution of HaSE1 and HaSE2 in other insect species

**Figure 4:**
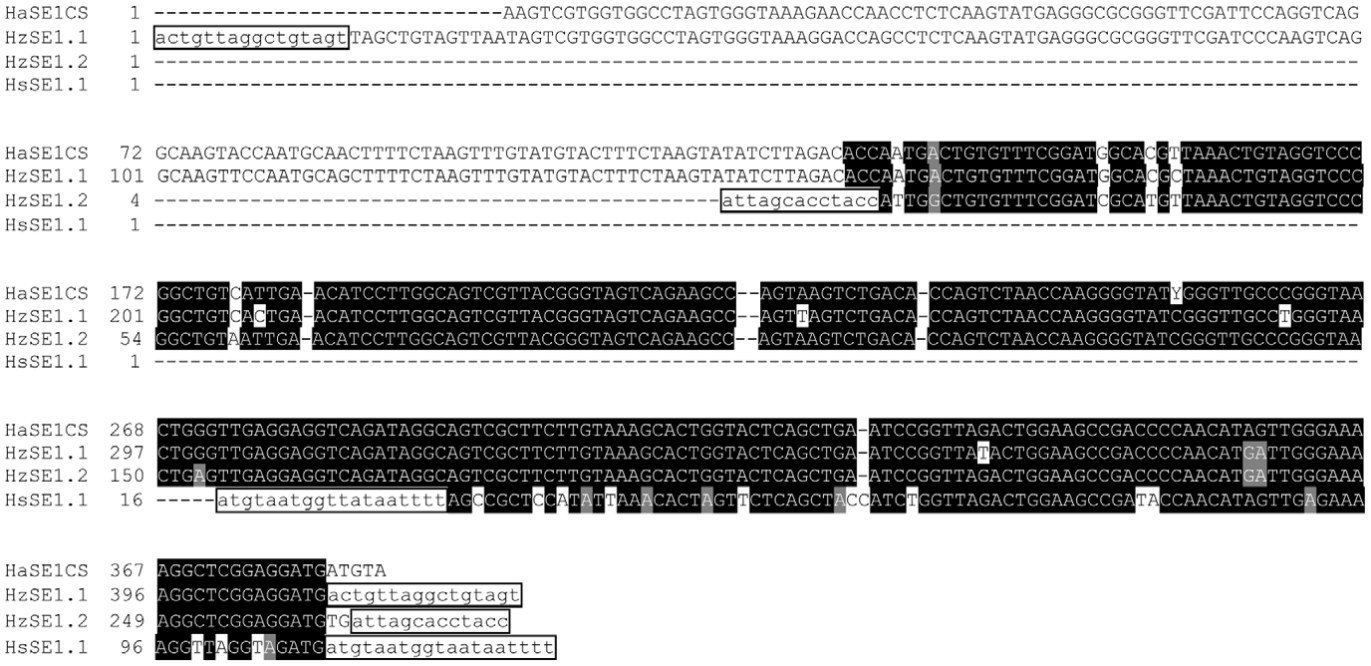
Alignments of selected sequences from GenBank entries sharing high identity with HaSE1. The sequence on the top line is the consensus sequence of the HaSE1 family. Putative flanking direct repeats are indicated in lowercase and boxed. Nucleotides shaded in black are conserved across sequences. These sequences were derived from the following GenBank entries: HzSE1.1 and HzSE1.2, DQ788840; HsSE1.1, GQ332573.

**Figure 5:**
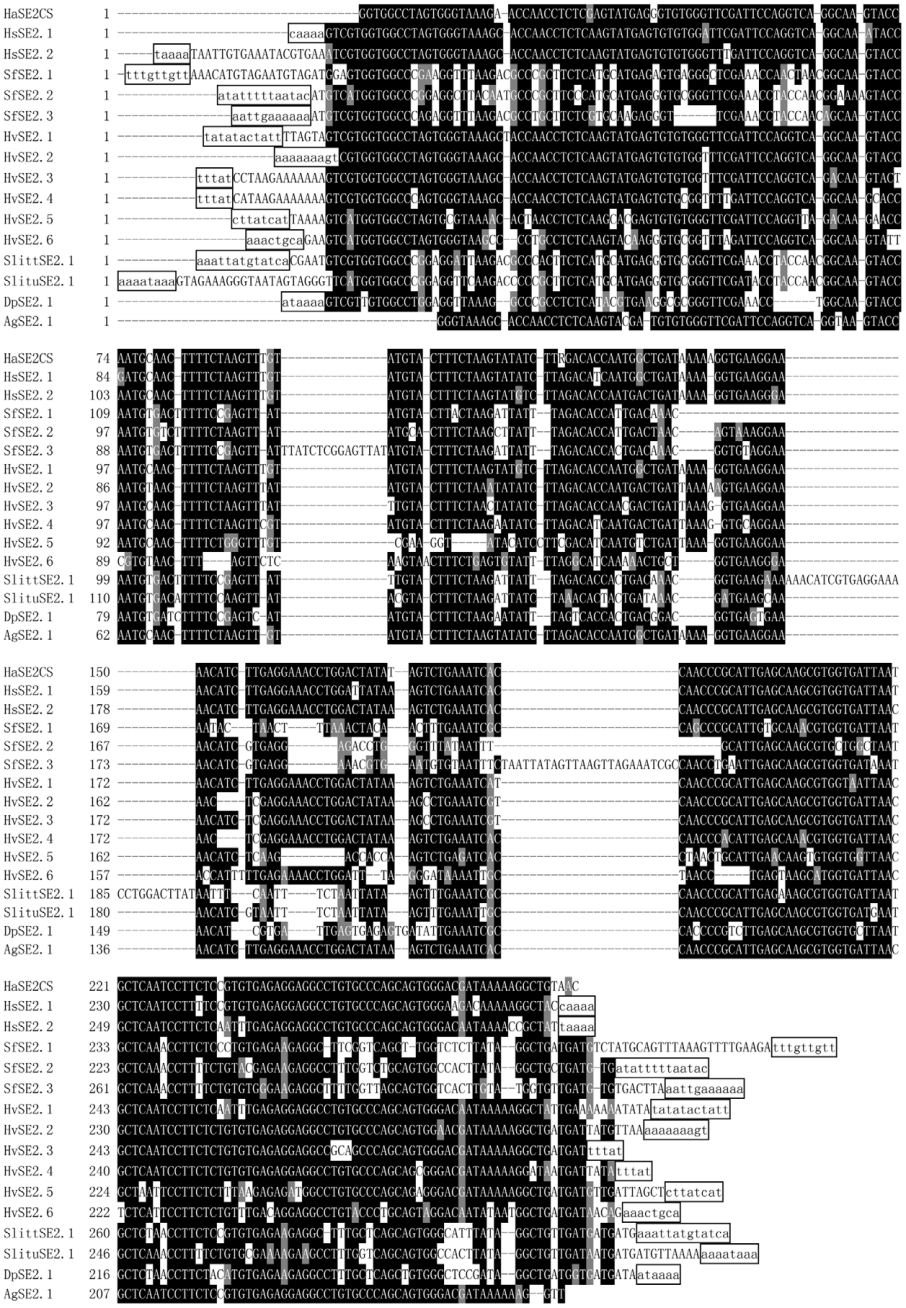
Alignments of selected sequences from GenBank and EST entries sharing high identity with HaSE2. The sequence on the top line is the consensus sequence of the HaSE2 family. Putative flanking direct repeats are indicated in lowercase and boxed. Nucleotides shaded in black are conserved across sequences. These sequences were derived from the following GenBank entries: HsSE2.1 and HsSE2.2, GQ332573; SfSE2.1, FP340417; SfSE2.2, FP340410; SfSE2.3, FP340416; HvSE2.1, GT188659; HvSE2.2, GT212998; HvSE2.3, HO703137; HvSE2.4, GR967973; HvSE2.5, GT132665; HvSE2.6, GT211688; SlittSE2.1, FQ015582; SlituSE2.1, GW413639; DpSE2.1, EY265072; AgSE2.1, GW550229.

All the above results suggested that HaSE1 and HaSE2 are two novel tRNA-derived SINEs. GenBank homology searches were further performed to detect HaSE1 and HaSE2 sequences in insect species other than *H. armigera*. Two copies of HaSE1-like elements including one full-length copy (named as HzSE1.1) and one 5′-truncated copy (HzSE1.2) were identified in the first and seventh introns of one cytochrome P450 gene, *CYP9A14*, in *H. zea* (accession number: DQ788840) (Figure 4). These two SINE elements were previously recognized as TE-like elements HzIS1-1 and HzIS1-2, respectively [28]. In *Heliothis subflexa*, one 5′-truncated copy of HaSE1-like elements (HsSE1.1) was found in an intron of the ABC transporter family C protein gene (accession number: GQ332573) (Figure 4). By searching with concensus HaSE2 sequence as a query, two full length copies of HaSE2-like elements (HsSE2.1, HsSE2.2) were found in introns of the ABC transporter family C protein gene from *H. subflexa* (accession number: GQ332573). Three copies (SfSE2.1, SfSE2.2 and SfSE2.3) were also found in BAC clones of *Spodoptera frugiperda* (accession number: FP340417, FP340410 and FP340416) (Figure 5).

The HaSE2 consensus sequence was also used to search the EST database using blastn and 50, 14,7 and 5 matches were detected in *Heliothis virescens*, *Spodoptera littoralis*, *Spodoptera litura* and *Danaus plexippus*, respectively, with an E-value less than 1e-25; of which 30, 12, 7 and 5 matches were 150 bases or longer. Representative examples of these sequences with perfect direct repeats are shown in Figure 5. Interestingly, one 267 bp EST sequence from *Aphis gossypii* (accession number: GW550229) showed high identity (96%) with the HaSE2 consensus sequence.

**Figure 6:**
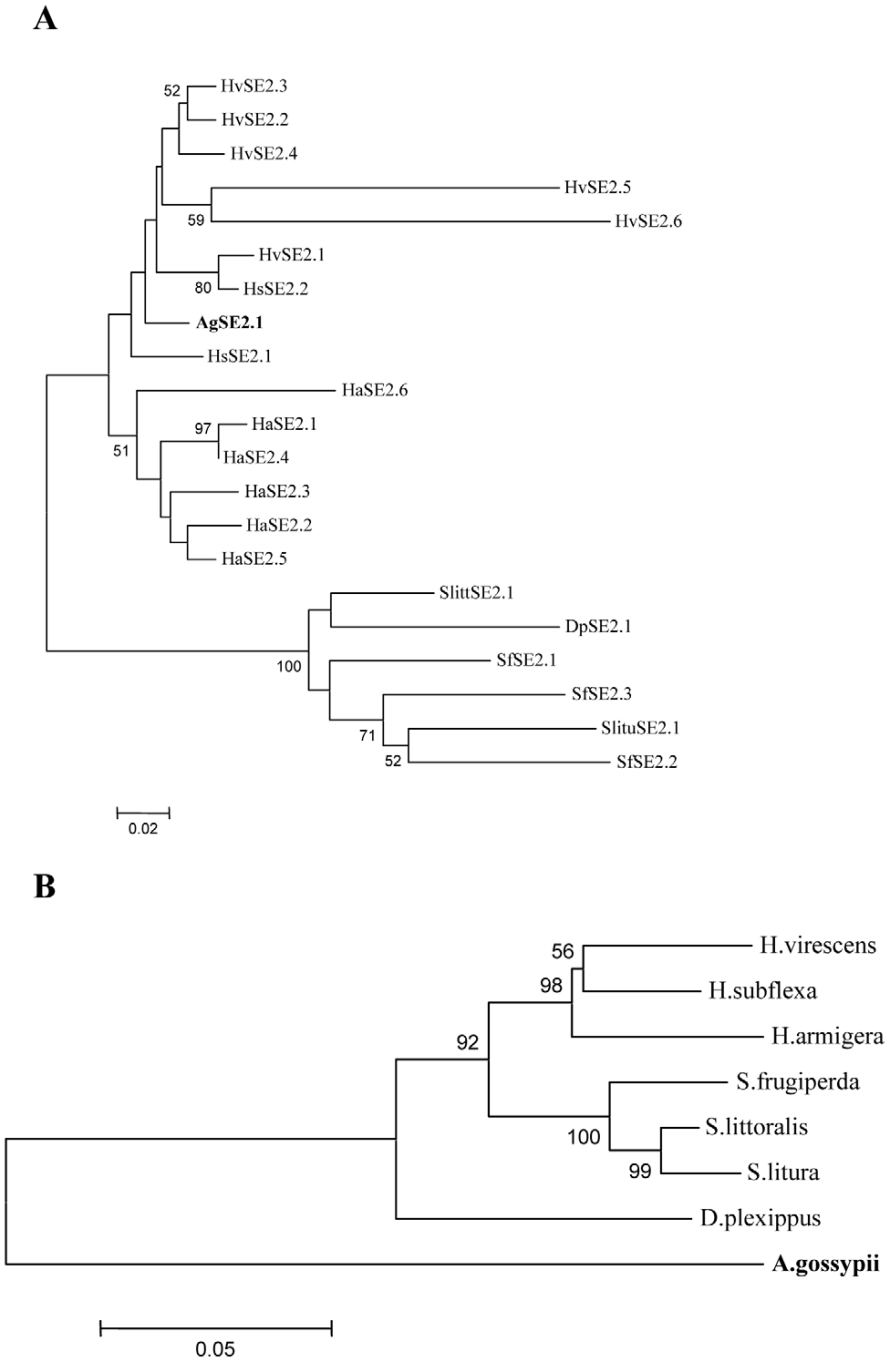
Phylogenetic analysis. (A) Phylogenetic relationships among HaSE2 elements in *Helicoverpa armigera* and similar elements in other insect species. (B) Phylogenetic relationships of *Helicoverpa armigera* and other insect species based on cytochrome oxidase subunit I (CO I) gene sequences. The Neighbor-joining tree was generated in MEGA5 with 1000 bootstrap replicates. Bootstrap values below 50% are not shown. CO I sequences are obtained from the following GenBank entries: JF415782 for *Helicoverpa armigera*, GU087832 for *Heliothis virescens*, EU768932 for *Heliothis subflexa*, GU090723 for *Spodoptera frugiperda*, FN908019 for *Spodoptera littoralis*, FN908025 for *Spodoptera litura*, DQ018954 for *Danaus plexippus*, EU701422 for *Aphis gossypii*.

The HaSE2 sequences were determined and analyzed for their evolutionary interrelationships with similar sequences from other insect species including *A. gossypii*. The results obtained by different phylogenetic methods were mostly congruent. We chose to present the topologies obtained by ML method (Figure 6). All the other trees obtained by the different methods are provided in Figure S4. The result indicates the existence of two major groups separated by a high bootstrap value of 100%. Elements from species of Heliothinae including *H. armigera*, *H. virescens* and *H. subflexa* together with one element from *A. gossypii* form a group, while elements from Amphipyrinae including *S. frugiperd*, *S. littoralis*, *S. litura* together with one element from Papilionoidea (*D. plexippus*) are clustered into the second group. Compared with the phylogenetic relationship deduced from cytochrome oxidase subunit I (CO I) gene sequences in these insect species, our data indicates that horizontal transfer might have occurred between heliothine species and *A. gossypii*.

### A 5S rRNA-derived SINE family in *Helicoverpa armigera* and related species

**Figure 7:**
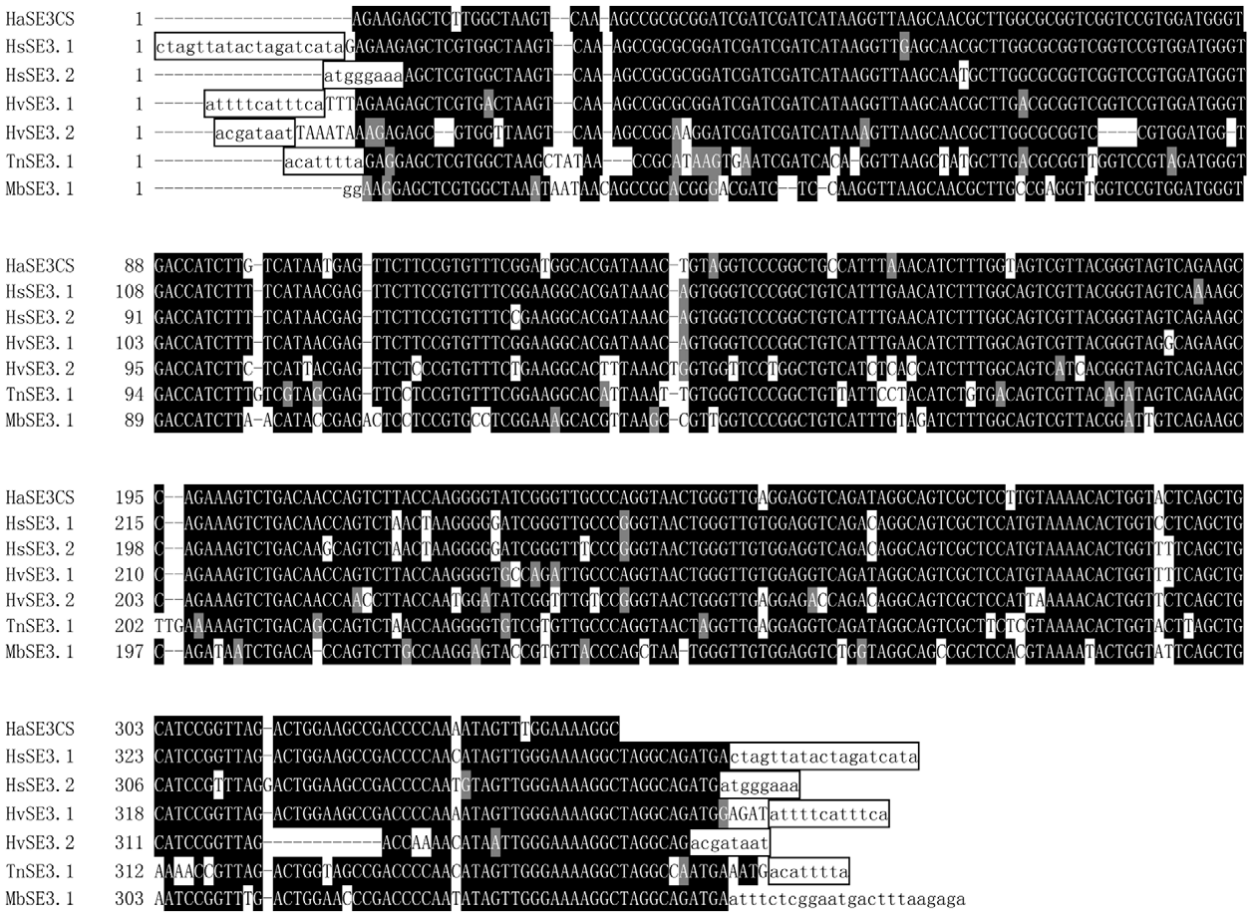
Alignments of the identified HaSE3 sequences in insect species other than *Helicoverpa armigera*. The sequence on the top line is the consensus sequence of the HaSE3 family. Putative flanking direct repeats are indicated in lowercase and boxed. Nucleotides shaded in black are conserved across sequences. GenBank entries for these sequences were shown in the text.

Using 246-bp 3′-region of HaSE1 as a query, our additional search has identified one putative repeat sequence in an intron of the diapause hormone-pheromone biosynthesis gene from *H. armigera* (accession number: AY382615) as well as two putative repeat sequences in introns of the ABC transporter family C protein gene from *H. subflexa* (accession number: GQ332573). Further analysis showed that the 5′-regions of these putative repeat sequences were most similar to 5S ribosomal RNA in *B. mori* (Accession number: K03316), suggesting these were 5S rRNA-derived SINEs. These sequences were named as HaSE3.1, HsSE3.1 and HsSE3.2, respectively (Figure S5, Figure 7). While the HsSE3.1 and HsSE3.2 were flanked by 19 bp and 8 bp TSDs, respectively, no TSDs were detected in HaSE3.1.

The HsSE3.1 sequence was used to search against the EST database using blastn. Two full length copies in *H. virescens* (accession number: GR960594 and GT199485) and one copy in *Trichoplusia ni* (accession number: CF259287) and *Mamestra brassicae* (accession number: FS939884) were identified, and were named as HvSE3.1, HvSE3.2, TnSE3.1and MbSE3.1, respectively (Figure 7).

For more comprehensive survey of the HaSE3 elements in *H. armigera*, we performed PCR using genomic DNA as template and two oligo-nucleotide primers that were specific for the 5S rRNA-related region and 3′ -LINE-related region, respectively (Table S1). A total of 15 different repeat sequences were obtained, and named as HaSE3.2–HaSE3.16 (Figure S5). Comparative analysis showed that the 3′ -region of HaSE3 is very similar to that of HaSE1 (94% identity between the two consensus sequences). The match between the HaSE3 elements and the consensus sequence ranged from 73% to 99%. The HaSE3.1 and HaSE3.16 showed notable differences with the other elements (only 73% and 80% identical to the HaSE3 consensus sequence, respectively), whereas the HaSE3.7, HaSE3.8 and HaSE3.9 are only 1% divergent from the HaSE3 consensus sequence.

## Discussion

### Conserved central domain in two tRNA-derived SINE families

Since the first discovery of rodent B1 and B2 elements as well as primate Alu elements about 30 years ago, more than 120 SINE families have been isolated from the genomes of mammals, reptiles, fishes, mollusks, ascidia, flowering plants, and insects [3], [40]. The majority of SINEs in eukaryotic genomes characterized to date are derived from tRNAs. They generally have a composite structure comprising a 5′ terminal tRNA-related region, which is followed by a non-tRNA-related region. In this study, we have identified two tRNA-derived SINE families, HaSE1 and HaSE2. Apart from the high identity between tRNA-related regions in their 5′ -regions, the following 62 bp regions also showed high identity. The finding of a conserved central domain in part of the body shared by distant SINE families is not surprising. To date, four such domains have been described: CORE domain in vertebrates [41], V-domain in fishes [42], Deu-domain in deuterostomes [10] and Ceph-domain in cephalopods [43]. The sequence identity between the conserved central domains of the HaSE1 and HaSE2 consensus sequences is 96.8%, whereas that of their tRNA-related regions is 95.7%. Such a high level of sequence identity between the conserved central domains of the two different SINE families suggests that the conserved central domain has been under strong selective constraint, most likely because it is functionally important for their retropositions.

### HaSE2, the first SINE family with its 3′ -region derived from DNA transposon

While the 5′ -structure of HaSE2 family shares high similarity with HaSE1, this SINE family has some features that distinguish it from the canonical SINEs. For instance, we did not detect a poly(A) tail or short direct repeats at the 3′ end of the HaSE2 elements, while conserved purine-rich ATAAAAA sequence was observed near the 3′ end. SINEs lacking a poly(A) tail have been found in most mammalian genomes and probably utilize other mechanisms for interacting with reverse transcriptase during retroposition [44]–[45].

Quite notably, the 154 bp 3′ -region showed high identity with one *mariner*-like element *Hamar1.3* in *H. armigera* [39]. To the best of our knowledge, it is the first report of a SINE family with its 3′ -region derived from DNA transposon. It is quite likely that HaSE2 elements resulted from integration of reverse transcriptases transcripts into the genomic copies of *mariner*-like elements or recombination of tRNA and transcripts of *mariner*-like elements during the template switching common for reverse transcriptases [46]–[47].

### HaSE3, a putative chimera of 5S rRNA and tRNA-derived SINE

Since Weiner predicted a new class of SINEs derived from 5S rRNA [48], such elements have been found in zebrafish and later in other fish species [9] and in a few mammals [10], fruit bats [11], and springhare [49]. Until recently, very little was known about the 5S rRNA-derived SINEs in insect genomes. Only one such repeat, SINE3-1_TC, has been found in the genome of the red flour beetle, *T. castaneum* [27]. In this study, the 5′-region of HaSE3 elements displays a considerable similarity with 5S rRNA, the highest similarity region was found in the promoter region composed of boxes A, IE, and C, indicating that HaSE3 is one novel 5S rRNA-derived SINE family in insects.

The body of SINEs is usually unique for each SINE family and its origin is largely obscure. Quite notably, the 3′ -region of HaSE3 including body part and 3′ tail are very similar to HaSE1. SINEs sharing a same 3′ -tail are not unique for HaSE1 and HaSE3. For example, an approximately 70-bp-long conserved 3′-tail were conserved in three SINE families including SImI, HpaI and OS-SINE1, which exhibits a significant homology to the 3′ -tail of the salmonoid RSg-1 LINE [50]. In this study, the high homology between HaSE3 and HaSE1 in 3′ -region suggested that the novel HaSE3 family might be generated by template switching from the tRNA-derived HaSE1 RNA to 5S rRNA during the process of cDNA synthesis in retroposition [10]. Thus, our study may represent the first description of a chimera of a 5S rRNA and a tRNA-derived SINE in insect species.

### Partner LINE

Unlike DNA transposons, integration of new copies of SINE into the new genomic locations occurs via a mechanism of reverse transcription, which relies entirely on the enzymatic machinery of autonomous partner LINE in the same genome. Recognition of SINE transcripts by LINE reverse transcriptase (RT) is guaranteed either by the common 3′ -tail shared by the SINE and its partner LINE (stringent recognition) or by the presence of the poly(A) tail (relaxed recognition ) [51]–[52].

Until recently, little was known about LINEs in *H. armigera*. Several LINE elements were identified in the first intron of cadherin gene and several microsatellites clones [53] as well as BAC clones [31]. In the present study, we identified one repeat element HaRTE1.1 in the novel LINE family, HaRTE1, in *H. armigera*. The 3′ -terminals of HaSE1 and HaSE3 consensus sequences were very similar to the very 3′ end of HaRTE1.1, suggesting that the 3′ ends of HaSE1 and HaSE3 descend from the 3′ end of the partner HaRTE1, and transpositions of HaSE1 and HaSE3 depend on the enzymatic machinery encoded by HaRTE1 element.

### Abundance and distribution of SINE families in *H. armigera*

The number of copies of SINEs varies from family to family. In *H. armigera*, assuming that the BAC contigs are representative and a genome size for *H. armigera* of 400 Mb [54], then a total of 11, 000 copies of HaSE1 and 1, 200 copies of HaSE2 would be predicted. We did not find any HaSE3 in BAC clones, thus the abundance of HaSE3 seems much lower than that of HaSE1 and HaSE2. The variety of copy numbers among these three SINE families is probably because of the differences of retroposition efficiency. The lacking of a poly(A) tail or short direct repeats at the 3′ end of HaSE2 might contribute to its relatively low retroposition efficiency and copy number. On the other hand, while HaSE3 shares the same 3′ end with HaSE1, their promoters might have quite different transcriptional activities. As proposed by Kapitonov and Jurka [9], the type 1 promoters in 5S rRNAs depend much more on upstream signals than do type 2 promoters in tRNAs. As a result, the Pol III promoter in a retroposed 5S rRNA copy presumably remains silent or is expressed at a low level.

SINEs are generally thought to be transmitted vertically from parents to offspring, and the probability of independent emergence of the same SINE families in unrelated species is negligible. Thus, the species sharing same SINE family or a SINE inserted in the same locus are considered as related. Consistent with this notion, our searches against various GenBank databases revealed the presence of HaSE1 in *H. zea* and *H. subflexa*, two closely-related species of *H. armigera*. Likewise, HaSE2 were found in *H. subflexa*, *H. virescens*, *S. frugiperd*, *S. littoralis*, *S. litura* and *D. plexippus*, and HaSE3 were found in *H. subflexa*, *H. virescens*, *T. ni* and *M. brassicae*. However, both the HaSE2 and HaSE3 SINEs were not found in *H. zea*, which is the closest relative of *H. armigera* and is thought to be derived from a founder population of *H. armigera* approximately 1.5 million years ago [55]. This is possibly because of the current limited availability of *H. zea* sequence, rather than the true absence of the two SINE families in the *H. zea* genome. More extensive investigation of the distribution of SINEs described in this paper would be very useful in phylogeny analysis of large and diverse families in Lepidopterans.

The fact that we did not find all the three SINE families in the fully-sequenced *B. mori* suggests that these three SINE families are probably narrowly distributed in subgroups of Lepidopterans. Thus, our finding of one HaSE2 element from the *A. gossypii*, a non-lepidopteran species, suggests the occurrence of horizontal transfer of HaSE2. This is not unusual as intensive horizontal transfer was observed for salmonid retroposons to the schistosome genomes [50], [56]. A recent study shows that poxviruses are possible vectors for the horizontal transfer of Sauria SINE from reptiles to mammals [57]. It is possible that some disease pathogens or natural enemies (e.g. parasitoids) that infect or attack both heliothine species and *A. gossypii* may serve as the vectors for the horizontal transfer of HaSE2. Further research is necessary to test this possibility and to identify the possible vectors.

## Supporting Information

Figure S1 **Alignments of the identified full length HaSE1 sequences in** ***Helicoverpa armigera*** **.** The sequence on the top line is the consensus sequence of the HaSE1 family. Putative flanking direct repeats are indicated in lowercase and boxed. Nucleotides shaded in black are conserved across sequences.
(RTF)

Figure S2 **Predicted secondary structure of HaSE1.** Secondary structure of the tRNA-related region in consensus sequence of HaSE1 was predicted by tRNAscan-SE.
(RTF)

Figure S3 **Alignments of the identified HaSE2 sequences in** ***Helicoverpa armigera*** **.** The sequence on the top line is the consensus sequence of the HaSE2 family. Putative flanking direct repeats are indicated in lowercase and boxed. No flanking direct repeats were found in HaSE2.6 and HaSE2.7. Nucleotides shaded in black are conserved across sequences.
(RTF)

Figure S4 **Phylogenetic analysis using maximum parsimony and Bayesian methods.** (A) Maximum parsimony tree for HaSE2 elements in *Helicoverpa armigera* and similar elements in other insect species. (B) Bayesian tree for HaSE2 elements in *Helicoverpa armigera* and similar elements in other insect species.
(DOC)

Figure S5 **Alignments of the identified HaSE3 sequences in** ***Helicoverpa armigera*** **.** The sequence on the top line is the consensus sequence of the HaSE3 family. Nucleotides shaded in black are conserved across sequences.
(RTF)

Table S1 **Primers used for genome walking and PCR amplification of HaSE3 elements.**
(RTF)

Table S2 **Genes found in the flanking sequences of full length HaSE1 and HaSE2.**
(RTF)

Table S3 **Genes found in the flanking sequences of 5′-truncated HaSE1.**
(RTF)
