# Supplementary material for: Characterization of Three Novel SINE Families with Unusual Features in Helicoverpa armigera
Source: PLoS One. 2012 Feb 3;7(2):e31355. doi: 10.1371/journal.pone.0031355 (PMC3272025; doi:10.1371/journal.pone.0031355)
Supplement: Figure S1 — Alignments of the identified full length HaSE1 sequences in Helicoverpa armigera . The sequence on the top line is the consensus sequence of the HaSE1 family. Putative flanking direct repeats are indicated in lowercase and boxed. Nucleotides shaded in black are conserved across sequences. (RTF) [file pone.0031355.s001.rtf]

HaSE1CS     1 ------------------------------------AAGTCGTGGTGGCCTAGTGGGTAAA-GAACCAACCTCTCAAGTATG----AGGGCGCGGGTTCGATTCCA--GG
HaSE1.1     1 ------------------atgctattGAATGACAAAAAGTCGTGGTGGCCTAGTGGGTAAA-GAACCAACCTCTCAAGTATG----AGTGTCTGGATTCGATTCTA--GT
HaSE1.2     1 -----------------acgttcttacaaactttcAAAGTCGTGGTGGCCCAGTGGGCAAA-GAACCAACCTCTCAAGTATG----AGAGCGTGGGTTCGATTCCA--GG
HaSE1.3     1 --------------aggaactatggcacctgGAAAAAAGTCGTGGTGGCCTGGTGGGCCAA-GAATCAACCTCTCAAGTATG----AGGGCGCGGGTTCGATTCCA--GG
HaSE1.4     1 -----ggttacggaaaaAGAAGTCGTGGTGGCCTAGAAGTCGTGGTGGCCTAGTGGGCAAA-GAACCAAACTCTCGAGCATG----AGGGCGCGGGTTCGATTCCA--GG 
HaSE1.5     1 ---------------------gtcatccttacATCCATGTCATGGTGGCCTAGTAGGTAAA-GAACCAACCTCTCAAGTACATATTAGGGCACGGGTTCGATTCCA--GG 
HaSE1.6     1 --------------tatgccgagagtttAAAAAAAAAAGTCGTGGTGGCCTAGTGGGTAAA-GGACCAACCTC--AAGTATG----AGGGCGCGGGGTCGATCCCA--GG 
HaSE1.7     1 -----------------accttttatcaagtgaaAAAAGTCGTGGTGGCCTAGTGGGTAAA-GAACCAACTTCTCAAGTATA----AGGGCGCGGGTTCGATCCCA--GG 
HaSE1.8     1 atgatggccaaaaTCAAATTCTATTCGTCCGTGTAAAAGTCGTGGTGGCCTAGTGGGCAAA-GGACCAACCTCTCAAGTAAG----AGGGCGCGGGTTCGATCCCA--GG
HaSE1.9     1 --------------------atagtaccgaagaaGAGAGTCGTGGTGGCCTAGTGGGTAAA-GGACCAGCCTCTCAAGTATG----AGGGCGCGGGTTCGATCCCA--GG 
HaSE1.10    1 --------------------------------tgggaTGGATCGGTGACCTAG-AGGTAAA-GAACCAAACTCTCAAGTATG----ATTG------TTCGATTCTA--GG 
HaSE1.11    1 ------------------atgtttcGAATTATTTTGAAGTCGTAGTGGAATAGTGGATAAAAGAATCAATCTCTTAAGTATG----AGTGCTTGGGTTTGAATCCA--GG 
HaSE1.12    1 ---------------attgacgaaaactattttGAAAAGTCGTGGTGGCCTAGTGGGTAAA-GGACCAACCTC--AAGTATG----AGGGCGCGGGGTCGATCCCA--GG 
HaSE1.13    1 -----------------acttcaaattgtaatatcGTGGTCGTGGTGGCCTAGTGGGTAAA-GGACCAACCTCTCAAGTATG----AGGGCGCGGGTTCGATCCCA--GG
HaSE1.14    1 -------------------aaataagtagcgttCGGGAGTCATGGTGGCCTAGTGGGCAAA-GAACCAATCTCTCGAGTATG----AGGGCGCGGGTTCGATTCCA--GG
HaSE1.15    1 --------------tgattgaaatgttaaTGTTAAAAAGTCGTGGTGGCCTAGTGGGTAAA-GGACCAACCTC--AAGTACG----AGGGCGCGGGTTCGATCCCA--GG
HaSE1.16    1 --------------------acagagtcgagggaaAAAGTCGTGGTGGCCTAGTGGGTAAA-GAACCAACCTCTCGAGTATG----AGGGCGCGGGTTCGATTCCA--GG
HaSE1.17    1 -------------------------attttgAAAGACCATCGTGGCGGCCTAGTGAGTAAA-GAGTCAACCTCTCGAGTACG----AGGGCGTTGGTTCGATTCCA--AG
HaSE1.18    1 ------------gacaaAGTCGTGATGACTTAGTGGAAGTCGTGGTGGTTTAGTGGGTAGA-GAACCAATCTCTCAAGTATG----AGCGTGCGTCTTTGATTCCA--GA
HaSE1.19    1 ---------------------atgaatgTAAGTACCTAGTAGTAGTG-CCTAGTGGGTAAA-GGACCAACCTC--AAGTATG----AGGGCGCGGGGTCGATCCCA--GG
HaSE1.20    1 ----------------aaaccgTTGTGATAGGTCTATCTTCGTAG-GGCCTAGTGGG-AAA-GAACCAACCTCTCAAGTATG----AGTGCGTGG-TTCGATTCCA--GG
HaSE1.21    1 ------------------------tataaTCACACTTTTTACTCTAGGCCTAGTGGGTAAA-GAACCAACCTCTCAAGTATA----AGTGCGTAAGTTCGGTTACGTCGC
HaSE1.22    1 ---------------------------tggccGCGGAAGTCGTGGTGGCCTAGTGGGTAAA-GGACCAACCTC--AAGTACG----AGGGCGCGGGTTCGATCCCA--GG


HaSE1CS    69 CAGGCAAGTACC----AATGCAACTTTTCTAAGTTTGTA---TGTA----CTTTCTAAGTATATCTTA----GACACCAATGACTGTGTTTCGG------ATGGCACGTT
HaSE1.1    86 TCAGGACAGTTTC-------------------------------GTA------------GTATATCTTG----GACACCTATGACTGAATTTCAG-----AGGGCATGTT
HaSE1.2    87 TCAGG-AAGTACC----AATGCAACATTTCTAAGTTTGTT---TGTA----CTTTCTAAGTATATC------------------------------------------TT
HaSE1.3    90 TCAGGCAAGTACC----AACGCAACTTTTCTAAGTTTGTA---TGTA----CTTTCTAAGTATATCCTCCAATGACACCAATGACTGTGTTTCGG-----ATGGCACGTT
HaSE1.4    99 TCAGGCAAGTACC----AATGCAACTTTTCCAAGTTTGTA---TGTA----CTTTCTAAGTATATCTTA----GACACCAATGACTGTGTTTCGG-----ATGGCACGTT
HaSE1.5    87 TCAGGCAAGTACC----AATGCAACTTTTTAAAGTTTATA---TGTA----CTTTCTAAGTATATCTTA----GACACCAATGACTGTGATTCGG-----ATGGCACGTT
HaSE1.6    88 TCAGGCAAGTACC----AATGCAACTTTTCTAAGTTTGTA---TGTA----CTTTCTAAGTATATCTTA----GACACCATTGACTGTGTTTCGG-----ATGGCACGTT
HaSE1.7    87 TCAGGCAAGTACC----AATGCAACTTTTCTAAGTT-GTA---TGTA----CTTTCTAAGTATTTCTTA----GACACCAATGACTGTGTTTCGG-----ATAGCACGTT
HaSE1.8   104 TCAGGCAAGTACC----AATGCAACTTTCC-AAGTTTGTA---TGTA----CTTTCTAAGTATATCTTA----GACACCATTGACTGTGTTTCGG-----ATGGCACGTT
HaSE1.9    84 TCAGGCAAGTACC----AATGCAACTTTTCTAAGTTTGTA---TGTA----CTTTCTAAGTATATCTTA----GACACCAATGACTGTGTTTCGG-----ATGGCGCGTT
HaSE1.10   65 TCAGGCAAGTACC----AATGCAACTTTTCTAAGTAT--------------------------ATCTTG----GACACTAATGACTGTGTT-CGA---CAA----ACGTT
HaSE1.11   87 CCAGGCAAGTACC----AATGCAACTTT---------GTA---TGTA----CTTTTTAAGTAGACTGTG------TTTCG--GACGGT-----------------ATGTT
HaSE1.12   87 TCAGGCAAGTACC----AATGCAACTTTTCTAAGTTTGTA---TGTA----CTTTCTAAGTATATCTTA----GACACCATTGACTGTGTTTCGG-----ATGGCACGTT
HaSE1.13   87 TCAGGCAAGTACC----AATGCAACTTTTCTAAGTTTGTA---TGTA----CTTTCTAAGTATATCTTA----GACACCATTGGCTGTGTTTCGG-----ATGGCACGTT
HaSE1.14   85 TCAGGCAAGTACC----AATGCAACTTTTCTAAGTTTGTA---TGTA----CTTTCTAAGTATATCTTA----GACGCCAATGGCTGTGTTTCGG-----ATGGCACGTT
HaSE1.15   88 TCAGGCAAGTACC----AATGCAACTTTTCTAAGTTTGTA---TGTA----CTTTCTAAGTATATCTTA----GACACCATTGGCTGTGTTTCGG-----ATGGCACGTT
HaSE1.16   84 TCAGGAAAGTACC----AATGCAACTTTTCTAAGTTTGTA---TGTA----CTTTCTAAGTATATCTTA----GACACCAATGACTGTGTTTCGG-----ATGGCACGTT
HaSE1.17   79 TCAGGCAAGTTCC----AATGCAAATTTTCTAAGTTTGTA---TGTA----CTTTCTAAGTAT-TCTTG----GACACCAATGACTGTGTTTCGG-----ATGACACGTT
HaSE1.18   92 TCTGGCAAATACCTGCTAATGCAACTTTTCTAAGTTCGTA---TTTT----CTTTCTACGTATATTTTG----GATACCAATGACCGTTATTTAG-----AGGGGATGTT
HaSE1.19   80 TCAGGCAAGTACC----AATGCAACTTTTCTAAGTTTGTA---TGTA----CTTTCTAAGTATATCTTA----GACACCATTAACTGTGTTTCGG-----ATGGCACGTT
HaSE1.20   85 TCAGGCAAGAGCC----AATGCAACTTTTCTAAGTTTGTA---TGTA----CTTTCTAAGTATATCTTG----GACACCAATGACTGT----------------------
HaSE1.21   82 TCGGGCAAGTACC----AATGCATCTTTTTTAAGCTTGTAACATGTC----CCTTCTAAGAAAATCATG----GGCACCAATGACTGTGTTTCGGATGCGATGGCACGTT
HaSE1.22   75 TCAGGCAAGTACC----AATGCAACTTTTCTAAGTTTGTA---TGTA----CTTTCTAAGTATATCTTA----GACACCATTGGCTGTGTTTCGG-----ATGGCACGTT


HaSE1CS   158 AAACTGTAGGTCCCGGCTGTCATT----GAACATCCTTGGC-AGTCGTTACGGG----------------------------------TAGTCAGAAGCCAGTAAGTCTG
HaSE1.1   144 AAACTGTAAGTCCCGGCTGTCAA-----GAACATCATCGGC-AGTCATTACGGG----------------------------------TAGT------------------
HaSE1.2   143 AGAC-----------ACT--------------------------------------------------------------------------------------------
HaSE1.3   184 AGACTGTAGGTCCCGGCTATCCTT----GAACATCCTTGGC-AGTCGTTACGGG----------------------------------TAGTCAAAAGTCAGTAAGTCTC
HaSE1.4   189 AAACTGTAGGTCCCGGCTGTCATT----GAACATCCTTGGC-AGTCGTTACGGG----------------------------------TAGTCAGAAGCCAGTAAGTCTG
HaSE1.5   177 AAACTGTAGGTCCCGGATGTCAAT----GAACTTCCTTGAC-AGTCGTTACGGG----------------------------------TAGTCAGAAGCCAGTAAGTCTG
HaSE1.6   178 AAACTGTAGGTCCCGGCTGTCAGT----GAACATCCTTGGC-AGTCGTTACGGG----------------------------------TAGTCAGAAGCCAGAAAGTCTG
HaSE1.7   176 AAACTGTAGGTCCCGACTGTCATT----GAACATCCTTGGC-AGTCGTTACGGG----------------------------------TGGTCAGAAGCCAGTAAATCTG
HaSE1.8   193 AAACTGTAGGTCCTGGCTGTCATT----GAACATCCTTGGC-AGTCGTTACGGG----------------------------------TAGTCAGAAGCCAGTAAGTCTG
HaSE1.9   174 AAACTGTAGGTCCTGGGAGTCATT----GAACATCCTTGGC-AGTCGTTACGGG----------------------------------TAGTCAGAAGCCAG-AAGTCTG
HaSE1.10  133 AAACTGTAGGTTTCGACTGGCATT----TATCATCCTTAGC-AGTCGTTACGCGTAGTCAGAGCCAGTAAGTCTGACACCAGTCTTACCAAGGGGTATTGGGT-------
HaSE1.11  152 AAACT----ATTTCAGCTATCATTCATTGAACATCCTTGGC-AGTCATTACGCG----------------------------------TAGTCAAAAGACTGTAGGTCTG
HaSE1.12  177 AAACTGTAGGTCCCGGCTGTCAGT----GAACATCCTTGGC-AGTCGTTACGGG----------------------------------TAGTCAGAAGCCAGAAAGTCTG
HaSE1.13  177 AAACTGTAGGTCCCGGCTGTCATT----GAACATCCTTGGC-AGTCGTTACGGG----------------------------------TAGTCAGAAGCCAGTAAGTCTG
HaSE1.14  175 AAACTGTAGGTCCCGGCTGTCATT----GAACATCCTTGGC-AGTCGTTACGGG----------------------------------TAGTCAGAAGCCAGTAAGTCTG
HaSE1.15  178 AAACTGTAGGTCCCGGCTGTCATT----GAACATCCTTGGC-AGTCGTTACGGT----------------------------------TAGTCAGAAGCCAGTAAGTCTG
HaSE1.16  174 AAACTGTAGGTCCCAGCTGTCATT----GAACATCCTTGGC-AGTCGTTACGGG----------------------------------TAGTCAGAAGCCAGTAAGTCTG
HaSE1.17  168 AAACCGTAGGTCCCGGCTGTCATT----GAACATCCTCGGT-AGTCGTTACGGG----------------------------------TAGTCAGAAGCCAGTAAGTCTG
HaSE1.18  186 AAACTGTAGGTTCCGGCTGTCATT----GAACAGTCTTGGC-AGTCGTTATGGG----------------------------------TAGTCAGAAGCCAGTAAGTCTG
HaSE1.19  170 AAACTGTAGGTCCCGGCTGTCAGT----GAACATCCTTGGC-AGTCGTTACGGG----------------------------------TAGTCAGAAGCCAGAAAGTCTG
HaSE1.20  158 ---------GTCCCGGCTGTCATT----GAACATCCATGGC-AGTTGTTACAGG----------------------------------TAGTCAGAAACCAGTAAGTCGG
HaSE1.21  180 AAACTATAGGTCCCGGCTGTCATT----GAACATCCTAGGC-AGTCGTTACGGG----------------------------------CAGTCAGAAGCCAGCAATTCTG
HaSE1.22  165 AAACTGTAGGTCCCGGCTGTCATT----GAACATCCTTGGC-AGTCGTTACGGG----------------------------------TAGTCAGAAGCCAGTAAATCTG


HaSE1CS   229 ACA-CCAGTCTAACCAAGGGGTATYGGGTTGCCCGGGTAACTGGGTTGAGGAGGTCAGATAGG-CAGTCGCTTCTTGTAAAGCACTGGTACTCAGCTGAATCCGGTTAGA
HaSE1.1   196 ACAACCAGTGTTAC-----GG----GGGTTGCCAGGGTAACCGAGT---------CAGATAGG-CAGTAGTTCCTTGTAAAGCACTGCTTCTCAGCTG----CGGTTAAA
HaSE1.2   150 -------------------GGTATTGG-TTGCCCGGGTAACTAGGTTGAGGAGGTCAGATAGGGCAGTAGCTCCTTGTAAACCACTGGTACTCAGCAGCATCCGGTTAGA
HaSE1.3   254 ACA-CCAGTCTAACCAAGGGGTATTGGGTTGCCTGGGTAACTGGGTTGAGGAGGTCAGATAGG-CAGTCGCTTCTTGTAAAGTACTGGTACTCGACTGAATCCGGTTAGA
HaSE1.4   260 ACA-CCAGTCTAACCAAAGGGTATCGGGTTGCCCGGGTAACTGGGTTGAGGAGGTCAGATAGG-CAGTCGCTTCTTGTAAAGAACTGGTACTCAGCTAAATCCGGTTAGA
HaSE1.5   248 ACA-CCAGTCTAACCAAGGGCTATTGGGTTGCCCGTTAAACTGGGTTCAGGAGGTCAGATAGG-CAGTCACTTGTTGTAAAGCACTG-TACTCAGCTGAATCCGGTTAGA
HaSE1.6   249 ACA-CCAGTCTAACCAAGGGGTATCGGGTTGCCCGGGTTACTGGGTTGAGGAGGTCAGATAGG-CAGTCGCTTCTTGTAAAGCACTGGTACTCAGCTGACTCGGGTTAGA
HaSE1.7   247 ACA-CCAGTCTAACCAAGGGGTATTGGGTCGCCCGGGTAACTGGGTTGAGGAGGTCAGATAGG-CAGTCGCTTCTTGTAAAGCAACGGTACTCAGCTGAATCCGGTTAGA
HaSE1.8   264 ACA-CCCGTCTAACCAAGGGGTATCGGGTTGCCCGGGTAACTGGGTTGAGGAGGTCAGATAGG-CA---GCTTCTTGTAAAGCACTGGTACTCAGCTGAATCCGGTTAGA
HaSE1.9   244 ACA-CCAGTCTAACCAAGGGGTATCGGGTTGCCTGGGTAACTGGGTTGAGGAGGTCAGACAGG-CAGTCGCTTCTTGTACAGCACTGATACTCAGCTGAATCCGGTTAGA
HaSE1.10  231 -----------------------------------TGC--CTGGGTTGAGGAGGTCAG---------TCGCTTCTTGTAAAACACTGGTACTCAGTTGCATCCGCTTAGA
HaSE1.11  223 CTAATCAATCTC-CCAAAAAGGATTGCTTTGCCCGGGAAACTGGGTTGAGGAGGTCAGATAGG-CAGTCGCTCCTTGTGAAACACTGGTACTCAACTGCATCCGGTAAGA
HaSE1.12  248 ACA-CCAGTCTAACCAAGGGGTATCGGGTTGCCCGGGTTACTGGGTTGAGGAGGTCAGATAGG-CAGTCGCTTCTTGTAAAGCACTGGTACTCAGCTGAATCCGGTTAGA
HaSE1.13  248 ACA-CCAGTCTAACCAAGGGGTATCGGGTTGCCCGGGCAACTGGGTTGAGGAGGTCAGATAGG-CAGTCGCTTCTTGTAAAGCACTGGTACTCAGCTGAATCCGGTTAGA
HaSE1.14  246 ACA-CCAGTCTATCCAAGGGGTATTGGGTTGCCCGGGTAACTGGGTTGAGGGGGTCAGATAGGGCAGTCGCTCCTTGTAAAGCACTGGTACTCAGCTACATCCGGTTAGA
HaSE1.15  249 ACA-CCAGTCTAACCAAGGGGTATCGGGTTGCCCGGGTTACTGGGTTGAGGAGGTCAGATAGG-CAGTCGCTTCTTGTAAAGCACTGGTACTCAGCTGAATCCGGTTAGA
HaSE1.16  245 ACA-CCAGTCTAACCAAGGGGTACCGAGTTGCCCGGGTAACTAGGTTGAGGAGGTCAGATAGG-CAGTCGCTTCTTGTAAAGCACTGGTACTCAGCTGAATCCGGTTAGA
HaSE1.17  239 ACG-CCAGTCTAACCAAGGGGTATTGGGTTGCCCGGGTAACTGGGTTAAGGAGGTCAGATAGG-CAGTCGCTCCTTTTAAACCCCTGGTACTCAGCTGAATCCGGTTAGA
HaSE1.18  257 AC---CAGTTTTACCAAGGGGTGTTGGGTTGAGCGGGTAACCGGGTTGTGGAGGTCAGATGGG-CAGTCGCTCCTTGTAAAACACTGGTACTCAGTTGCATC----GTGA
HaSE1.19  241 ACA-CCAGTCTAACCAAGGGGTATCGGGTTGCCCGGGTTACTGGGTTGAGGAGGTCAGATAGG-CAGTCGCTTCTTGTAAAGCACTGGTACTCAGCTGAATCCGGTTAGA
HaSE1.20  220 ACA-CCAGTATAACCAAGGGGTATTGGGTTGCCTGGGTAATTTGGTTG-------AAGACAGA-CAATCGCTC--TGGAAAGC-CTGG--CTC---------------GA
HaSE1.21  251 ACA------------------------------CCTGTAACTGGGTTGAGGAGGTCAGATAGG-CAGTCGCTCCTTGTAAAACACTGGTACTTAGCTGCATCCGGTGAGA
HaSE1.22  236 ACA-CCAGTCTAAGCAAGGGGTATCGGGTTGCCCGGGTAACTGGGTTGAGGAAGTCAGATAGGGCAGTCGCTTCTTGTAAAGCACTGGTACTCAGCTGAATCCGGTTAGA


HaSE1CS   337 C-------TGGAAGCCGACCCCAACAT--------AGTT-GGGAAAA--GGCTCGGAGGATGATGATG
HaSE1.1   283 C-------TGTAAGCCGACCCTAACAT--------AGTT-GGGAAAA--GGCTCGGGAGATGATGATGatgctatt
HaSE1.2   240 C-------TGGAAGCCGACCCCAGCAT--------AGTT-GGGAAAAA--GCTCGGAGGATGATG--GATGacgttcttacaaactttc
HaSE1.3   363 C-------TGGAAGCCGACCCCAACAT--------AGTT-GGGAAAAAAGGCTCGGAGGATGATGATGATGaggaactatggcacctg
HaSE1.4   368 C-------TGGAAGCCGACCCCAACAT--------AGTTTGGGAAAA--GGCTCGGAGGATGATggttacggaaaa
HaSE1.5   355 C-------TGGAAGCCGACCCCAACAT--------AGTT--GGAAAAAAGGCTCGGATGATGATGATGATGTgtcatccttac
HaSE1.6   357 C-------TGGAAGCCGACCCCAACGT--------GATT-GGGAAAA--GGCTCGGAGGATGATGAtatgccgagagttt
HaSE1.7   355 C-------TGGAAGCCGACCCCAACAT--------AGTT-GGGAAAAAAGGCTCGAAGGATGATGaccttttatcaagtgaa
HaSE1.8   369 C-------TGGAAGCCGACCCCAACAT--------GATT-GGGAAAA--GGCTCGGAGGATGatgatggccaaaa
HaSE1.9   352 C-------TGGAAGCCGAACCTAACGT--------GGTT-GGGAAAAA-GGCTCGGAGGATGatagtaccgaagaa
HaSE1.10  286 C-------TTCAAGCCGACCCCAACATACATATGAAGTT-GAGAAAAA-AGATGGG-AAATGATGAtggga
HaSE1.11  331 C-------TGGTAGCCGATCCTAACAT--------AGTT-GGGA-AAA-GGCTCGGGAAATTatgtttc
HaSE1.12  356 C-------TGGAAGCCGACCCCAACGT--------GATT-GGGAAAA--GGCTCGGAGGATGATGattgacgaaaactatttt
HaSE1.13  356 C-------TGGAAGCCGACCCCAACAT--------GATT-GGGAAAAA-GGCTCGGAGGATGATGATGacttcaaattgtaatatc
HaSE1.14  355 C-------TGGAAGCCGACCCCAACGT--------AGTTTGAGAAAAA-GGCTCGGAGGATGaaataagtagcgtt
HaSE1.15  357 C-------TGGAAGCCGACCCCAACAT--------GATT-GGGAAAA--GGCTCGGAGGATGATGAtgattgaaatgttaa
HaSE1.16  353 C-------TGGAAGCCGACCCCAACAT--------AGTT-GGGAAAA--GGCTCGGAGGATGacagagtcgagggaa
HaSE1.17  347 C-------AGGAAGCCGACCCCAACAA--------AGTT-GGGAAAA--GGCTCGGGAGATGattttg
HaSE1.18  359 C-------TGGAAGTCGACCCTAACAT--------AATT-GGGATAAA-GGCTCTTGAGATAATAACgacaa
HaSE1.19  349 C-------TGGAAGCCGACCCCAACGT--------GATT-GGGAAAA--GGCTCGGAGGATGatgatg
HaSE1.20  301 C-------TGGAAGCCGACCCCAACAT--------AGTA-AGGA-AAA-GGCTCGGGAGATGATGATGTTTTGGATCaaaccg
HaSE1.21  330 CGAGGAGCTGGGAGCCGACCCCAACAT--------AGTT-GGGAAAAG--GCTCGGCAGATAATGACGTCTAAGtagaa
HaSE1.22  345 C-------TGGAAGCCGACCCCAACAT--------GATT-GGGAAAA--GGCTCGGAGGATGATGAGGATGAtggtc

Figure S1. Alignments of the identified full length HaSE1 sequences in Helicoverpa armigera. The sequence on the top line is the consensus sequence of the HaSE1 family. Putative flanking direct repeats are indicated in lowercase and boxed. Nucleotides shaded in black are conserved across sequences.
