# Supplementary material for: Characterization of Three Novel SINE Families with Unusual Features in Helicoverpa armigera
Source: PLoS One. 2012 Feb 3;7(2):e31355. doi: 10.1371/journal.pone.0031355 (PMC3272025; doi:10.1371/journal.pone.0031355)
Supplement: Figure S2 — Predicted secondary structure of HaSE1. Secondary structure of the tRNA-related region in consensus sequence of HaSE1 was predicted by tRNAscan-SE. (RTF) [file pone.0031355.s002.rtf]

Figure S2. Predicted secondary structure of HaSE1. Secondary structure of the tRNA-related region in consensus sequence of HaSE1 was predicted by tRNAscan-SE.
