# Supplementary material for: Characterization of Three Novel SINE Families with Unusual Features in Helicoverpa armigera
Source: PLoS One. 2012 Feb 3;7(2):e31355. doi: 10.1371/journal.pone.0031355 (PMC3272025; doi:10.1371/journal.pone.0031355)
Supplement: Figure S3 — Alignments of the identified HaSE2 sequences in Helicoverpa armigera . The sequence on the top line is the consensus sequence of the HaSE2 family. Putative flanking direct repeats are indicated in lowercase and boxed. No flanking direct repeats were found in HaSE2.6 and HaSE2.7. Nucleotides shaded in black are conserved across sequences. (RTF) [file pone.0031355.s003.rtf]

HaSE2CS    1 ----------------------------------TGGTGGCCTAGTGGGTAAAGAACCAACCTCTCGAGTATGAGGGTGTGGGTTCGATTCCAGGTCAGGCAAGTACCAA
HaSE2.1    1 ------------------- tagataggaaatCGCGGTGGCCTAGTG--CAAAGAACCAACCTCTCGAGTATGAGGGCGCGGGTTCGAATCCAGGTCAGGCAAGTACCAA
HaSE2.2    1 -------------------ttttaaTGTGTGTCCTGGTGGCCTAGTGGGTAAAGAACCAACCTCTCGAGTATGAGGGTGTGGGTTCGATTCCAGTTCAGGCAAGTACCAA
HaSE2.3    1 ------------------cagaccGACTAAGTCATGGTGGCCTAGTGGGTGAAGAACCAACCTCTCGAGTATGAGGGTGTGGGTTCGATTCCAGTTCAGGCAAGT-----
HaSE2.4    1 --------------------gtggaGATCAAAGGGGGAGGCCTAGTGGGCAAAGAACCAACCTCTCGAGTATGAGGGCTCGGGTTCGAATCCAGGTCAGGCAAGTACCAA
HaSE2.5    1 -----------------------------agttgtGGTGGCCTAGTGGGTAAAGAACCAACCTCTCGAGTATGAGGGTGTGGGTTCGATTCCAGGTCAGGCAAGTACCAA
HaSE2.6    1 ttggtaagctatatagtttatttatttgtggtgtTGGTGGCCTAGTGGGTAAAGAACCAACCTCTCTAATATGAAGGTGTGGGTTCGATTCCAGGTCAGGCAAGTACCAA
HaSE2.7    1 taaggtattatgatgactagttaaaaaattgattaGATGGCCTACTGGGTAAATCACCAAGCTCCTAACTATGAGCTAGTAGATTCGATACCAGGTCA------------


HaSE2CS   77 TGCAACTTTTCTAAGTTTGTATGTACTTTCTAAGTATATCTTRGACACCAATGGCTGATAAAAAGGTGAAGGAAAACATCTTGAGGAAACCTGGACTATA----------
HaSE2.1   89 TGCAACTTTTCTAAGTTTGTATGTACTTTCTAAGTATATCTTAGACACCAATGGCTGATAAAAAGGTGAAGGAAAACATCTTGAGGAAACCTGGACTATA----------
HaSE2.2   92 TGCAACTTTTCTTAGTTTGTATGTACTTTCTAAGTATATCTTGGACACCAATGGCTGATAAAAAGGTGAAGGAAAACATCTTGAGGAAACCTGGACTATA----------
HaSE2.3   88 -GCAACTTTTCTAAGATTGTATGTACTTTCTTAGTATATCTTGGACACCGATGGCTGATAAAAAGGTGAAGGAAAACATCTTGAGGAAACCTGGACTATAGTCCAGTGTA
HaSE2.4   91 TGCAACTTTTCTAAGTTTGTATGTACTTTCTAAGTATATTTTAGACACCAATGGCTGATAAAAAGGTGAAGGAAAACATCTTGAGGAAACCTGGACTATA----------
HaSE2.5   82 TGCAACTTTTCTAAGTTTGTATGTACTTTCTAAGTATATCTTAGACACCAATGGCTGATATAAAGGTGAAGGAAAACATCTTGAGGAAACCTGGACTATT----------
HaSE2.6  111 TGCAACTTTTCTAAGTTTTTATGTACTTTCAAAGTATATCTTGGACACTAATGGCTGATAAAAAGTTGAAGGAAAACATCTTGAGGAAACCTGGACTACA----------
HaSE2.7   99 ----ACTTTTCTACGTGTGTATACACTTT-TAAG-ATATTTTGGATACCAAT------TA------------AAAGCATACTGAAACAA----------A----------


HaSE2CS  177 ---TAGTCTGAAATCACCAACCCGCATTGAGCAAGCGTGG-------TGATTAATGCTCAATCCTTCTCCGTGTGAGAGGAGGCCTGTGCCCAGCAGTGGGACGATAAAA
HaSE2.1  189 ---TAGTTTGAAATCACCAACCTACATTGAGCAAGCGTGG-------TGATTAATGCTCAATCCTTCTCCGCGTGAGAGGAGGCCTGTGCCCAGCAGTGGGACGATAAAA
HaSE2.2  192 ---TAGTCTGAAATCACCAACCCGCATTGAGCAAGCGTGG-------TGATTAATGCTCAATCCTTCTCCGTGTGAGAGGAGGCCTGTGCCAAGCAGTGAGACAATAAAA
HaSE2.3  197 GTATAGTCTGAAATCACCAACCCGCATTGAGCAAGCGTGGTA---GGTAATTAATGCTCAATCCTTCTCCGTGTGAGAGGAGGCCTGTACCCAGCAGTGGGACGATAAAA
HaSE2.4  191 ---TAGTCTGAAATCACCAACCCGCATTGAGCAAGCGTGG-------TGATTAATGCTCAATCCTTCTCCGTGTGAGAGGAGGCCTGTGCCCAGCAGTGGGACGATAAAT
HaSE2.5  182 ---AAGTCTGAAATCGCCAACCCGCAATGAGCAAGCGTGG-------TGATTAATGCTCAATCCTTCTCCATGTGAGAGGAGGCCTGTGCCAAGCAGTGGGACGATAAAA
HaSE2.6  211 ---AAGTCTGAAATCACCAGCCCGCATTGAGCAAGCATGGG------TTATTAATGCTTAATCCTTCTCTGTGTGAGAGGAGGATTGTGCCCAGCAGTGGAACGATAAAA
HaSE2.7  165 ----AGCCTGAAAGAGCCAACCCGCATAAAGCAAGGGTGGTGAATGGTGATTAATG-TCAATCCTTCTCTGTGTGAGACCAGGCCGCAGTCAAGCAG-AAGATGGTAAAA


HaSE2CS  277 A-GGCTG-TAAC
HaSE2.1  289 A-GGCTG-TAACTTAtagataggaaat
HaSE2.2  292 AAGGCTG-TAACAGATGttttaa
HaSE2.3  304 A-GGCTA-TAAcagacc
HaSE2.4  291 A-GGCTG-TAACTAACTATGTTCAGCAgtgga
HaSE2.5  282 A-GGCTG-TAACagttgt
HaSE2.6  312 A-GGCTG-TAACaacaacctcttaaaatgatctgacctccag
HaSE2.7  269 A-GACTGATAAGggtgatgctgaacaggaaaatttgttcaaa

Figure S3. Alignments of the identified HaSE2 sequences in Helicoverpa armigera. The sequence on the top line is the consensus sequence of the HaSE2 family. Putative flanking direct repeats are indicated in lowercase and boxed. No flanking direct repeats were found in HaSE2.6 and HaSE2.7. Nucleotides shaded in black are conserved across sequences.
