# Supplementary material for: Characterization of Three Novel SINE Families with Unusual Features in Helicoverpa armigera
Source: PLoS One. 2012 Feb 3;7(2):e31355. doi: 10.1371/journal.pone.0031355 (PMC3272025; doi:10.1371/journal.pone.0031355)
Supplement: Figure S4 — Phylogenetic analysis using maximum parsimony and Bayesian methods. (A) Maximum parsimony tree for HaSE2 elements in Helicoverpa armigera and similar elements in other insect species. (B) Bayesian tree for HaSE2 elements in Helicoverpa armigera and similar elements in other insect species. (DOC) [file pone.0031355.s004.doc]

**A**

**B**

**Figure S4. Phylogenetic analysis using maximum parsimony and Bayesian methods.** (A) Maximum parsimony tree for HaSE2 elements in *Helicoverpa armigera* and similar elements in other insect species. (B) Bayesian tree for HaSE2 elements in *Helicoverpa armigera* and similar elements in other insect species.
