# Supplementary material for: Characterization of Three Novel SINE Families with Unusual Features in Helicoverpa armigera
Source: PLoS One. 2012 Feb 3;7(2):e31355. doi: 10.1371/journal.pone.0031355 (PMC3272025; doi:10.1371/journal.pone.0031355)
Supplement: Figure S5 — Alignments of the identified HaSE3 sequences in Helicoverpa armigera . The sequence on the top line is the consensus sequence of the HaSE3 family. Nucleotides shaded in black are conserved across sequences. (RTF) [file pone.0031355.s005.rtf]

HaSE3CS     1 ------------------------AGA--AGAGCTCTTGGCTAAGTCAA-AGCCGCGCGGATCGATCGATCATA-AGGTTAAGCAACGCTTGGCGCGGTC
HaSE3.1     1 ATGGTAGCTGATAGTTTATTTTGAAGATGAGAGCTCTTGGCCAAGTAAC-AGCCGCAAGGGTCGATCAATAGTA-AGGTAAAGCAAGAATTGGAGCTACT
HaSE3.2     1 ------------------------AGA--AGAGCTCGTGGCTAAGTAAA-AGCCGCGCGGATCGATCGATCATA-AGGTTAAGCAACGCTTGGCGCGGTC
HaSE3.3     1 ------------------------AGA--AGAGCTCTTGGCTAAGTAAA-AGCCGCGCGGATCGATCGATCATA-AGGTTAAGCAACGCTTGGCGCGGTC
HaSE3.4     1 ------------------------AGA--AGAGCTCTTGGCTAAGTAAA-AGCCGCGCGGATCGATCGATCATA-AGGTTAAGCAACGCTTGGCGCGGTC
HaSE3.5     1 ------------------------AGA--AGAGCTCTTGACTAAGTCAA-AGCCGCGCGGATCGATCGATCATA-AGGTTAAGCAACGCTTGGCGCGGTC
HaSE3.6     1 ------------------------AGA--AGAGCTCTTGACTAAGTCAA-AGCCGCGCGGATCGATCGATCATA-AGGTTAAGCAACGCTTGGCGCGGTC
HaSE3.7     1 ------------------------AGA--AGAGCTCTTGGCTAAGTCAA-AGCCGCGCGGATCGATCGATCATA-AGGTTAAGCAACGCTTGGCGCGGTC
HaSE3.8     1 ------------------------AGA--AGAGCTCTTGGCTAAGTCAA-AGCCGCGCGGATCGATCGATCATA-AGGTTAAGCAACGCTTGGCGCGGTC
HaSE3.9     1 ------------------------AGA--AGAGCTCTTGGCTAAGTCAA-AGCCGCGCGGATCGATCGATCATA-AGGTTAAGCAACGCTTGGCGCGGTC
HaSE3.10    1 ------------------------AGA--AGAGCTCTTGGCTAAGTCAA-AGCCGCGCGGATCGATCGATCATA-AGGTTAAGCAACGCTTGGCGCGGTC
HaSE3.11    1 ------------------------AGA--AGAGCTCTTGACTAAGTTAAGAGCAGC-CGGATCGATCAATCATA-AGGTTAAGCAATGCTTGACGCGGTC
HaSE3.12    1 ------------------------AGA--AGAGCTCTTGGCCAAGTCAA-AGCATCAAATAAAACCCGATTATAAAAGTTAAGCAACGCTTGTAGCGGTC
HaSE3.13    1 ------------------------AGA--AGAGCTCTTGGCTAAGTCAA-AGCCG----AATCGACCGATCATA-AGGTTAAGCAACGCTTGGCGCGGCC
HaSE3.14    1 ------------------------AGA--AGAGCTCTTGACCAAGTCAT-A------------------------AGGTTAAGCAACACTTGGCGCTGTC
HaSE3.15    1 ------------------------AGA--AGAGCTCTTGGCCAAGTCAC-AGCCGTGGGTATCGATCGATCATA-AGGTTAAGCAACGCTTGGCGCGGTC
HaSE3.16    1 ------------------------AGA--AGAGCTCTTGGCTAAGTAAC-AGCCGCAAGGGTCGATCGGTTATA-AGATAGAGCAATAGTTGTCGCTGTT


HaSE3CS    73 GGTCCGTGGATGGGTGACCAT----CTTGTCATAATGAGTTCTTCCGTGTTTCGGATGGCACGATAAACTG-TAGGTCCCGGCTGCCATTTAAACATCTT
HaSE3.1    99 GATCTGTAGATGGGTGACCATGCATCTTATCATAATGAGTTCTGAC-TGTTTCGGAGAGCACGTTAAATTGGTGGGTACTGGTTGTCATACGAACAACTT
HaSE3.2    73 GGTCCGTGGATGGGTGACCAT----CTTATCATACTGAGTTCTTCCGTGTTTCGGATGGCACGATAAACTG-TAGGTCCCGGCTGCCATTTAAACATCTT
HaSE3.3    73 GGTCCGTGGATGGGTGACCAT----CTTATCATACTGAGTTCTTCCGTGTTTCGGATGGCACGATAAACTG-TAGGTCCCGGCTGCCATTTAAACATCTT
HaSE3.4    73 GGTCCGTGGATGGGTGACCAT----CTTGTCATAATGAGTTCTTCCGTGTTTCGGATGGCACGATAAACTG-TAGGTCCCGGCTGCCATTTAAACATCTT
HaSE3.5    73 GGTCCGTGGATGGGTGACCAT----CTTGTCATAACGAGTTCTTCCGTGTTTCGGATGGCACGATAAACTG-TAGGTCCCGGCTACCATTTAAACATCTT
HaSE3.6    73 GGTCCGTGGATGGGTGACCAT----CTTGTCATAATGAGTTCTTCCGTGTTTCGGATGGCACGATAAGCTG-CAGGTCCCGGCTGCCATTTAAACATCTT
HaSE3.7    73 GGTCCGTGGATGGGTGACCAT----CTTGTCATAATGAGTTCTTCCGTGTTTCGGATGGCACGATAAACTG-TAGGTCCCGGCTGCCATTTAAACATCTT
HaSE3.8    73 GGTCCGTGGATGGGTGACCAT----CTTGTCATAATGAGTTCTTCCGTGTTTCGGATGGCACGATAAACTG-TAGGTCCCGGCTGCCATTTAAACATCTT
HaSE3.9    73 GGTCCGTGGATGGGTGACCAT----CTTGTCATAATGAGTTCTTCCGTGTTTCGGATGGCACGATAAACTG-TAGGTCCCGGCTGCCATTTAAACATCTT
HaSE3.10   73 GGTCCGTGGATGGGTGACCAT----CTTGTCATAATGAGTTCTTCCGTGTTTCGAATGGCACGATAAACTG-TAGGTCCCGGCTGCCATTTGAACATCTT
HaSE3.11   73 GTTCGGTGGATGGGTGACCAT----CTTGTCTTGACGAGTATCTTCGTGTTACGGAAGGCACGTTAAA-----------TAGTTGTCATTTGAACATCTT
HaSE3.12   74 GGTCCTTGGATGGGTGACCAT----GTTGTCATGACCAGTTCATT-ATGTATC-------------------------CTGGCTATCATT-GAACATCTT
HaSE3.13   69 GGTCCATGGATCAGTGACCAT----CTTGTCATAATGAGTTCTTCCGTG--TCGAA-GGCACGATAAACTGGTGGGTCCCGGCTGTCTTTTTAACATCTT
HaSE3.14   50 AGTTCGTGGATGGGTGACCAT----CTAGTCATAACGAGTTCTTCCGTGTATCGGAAGGCACGATAAATTGTTGGGTCCCAGCTGTCATTTGAACATCTT
HaSE3.15   73 GGTCCGCGGATGGTTTACCAT----CTTGTCACAATAAGTTCATGCATGTTTCGGAAGGCGTGATAAATTGGTAGGTATCAGCTTTCATTTGAATGTCTT
HaSE3.16   73 GTCCTGT----GGGTGACCATGCATCTTATCATAATGAGTTCTGAC-T-TTTCGGAGGGCACGTTAAATTGGTAGGTGTCGGGTGTCATTTGAACATCTT


HaSE3CS   168 TGGTAGTCGTTACGGGTAGTCAGAAGCCAGAAAGTCTGACAACCAGTCTTACCAAGGGGTATCGGGTTGCCCAGGT-AACTGGGTTGAGGAGGTCAGATA
HaSE3.1   198 TTGCAATCTTTACGTGCAATCAGAATGCAGAAAGACAGATAAATAGTCTTAACAACGG--------TTGCCTGGGT-GACTGTGTTAAGCAGGTTACATA
HaSE3.2   168 TGGTAGTCTTTACGGGTAGTCAGAAGCCAGAAAGTCCGACATCCAGTCTTACCAAGGGGTATCGGGTTGCCCAGGT-AACTGGGTTGAGGAGGTCAGATA
HaSE3.3   168 TGGTAGTCTTTACGGGTAGTCAGAAGCCAGAAAGTCTGACAACCAGTCTTACCAAGGGGTATCGGGTTGCCCAGGT-AACTGGGTTGAGGAGGTCAGATA
HaSE3.4   168 TGGTAGTCGTTACGGGTAGTCAGAAGCCAGAAAGTCTGACATCCAGTCTTACCAAGGGGTATCGGGTTGCCCAGGT-AACTGGGTTGAGGAGGTCAGATA
HaSE3.5   168 TGGTAGTCGTTACGGGTAGTCAGAAGCCAGAAAGTCTGACAACCAGTCTTACCAAGGGGTATTGGGTTGCCCAGGT-AACTGGGTTGAGGAGGTCAGATA
HaSE3.6   168 TGGTAGTCGTTACGGGTAGTCAGAAGCCAGAAAGTCTGACAACCAGTCTTACCAAGGGGTATCGCGTTGCCCAGGT-AACTGGGTTGAGGAGGTCAGATA
HaSE3.7   168 TGGTAGTCGTTACGGGTAGTCAGAAGCCAGAAAGTCTGACAACCAGTCTTACCAAGGGGTATCGGGTTGCCCAGGT-AACTGGGTTGAGGAGGTCAGATA
HaSE3.8   168 TGGTAGTCGTTACGGGTAGTCAGAAGCCAGAAAGTCTGACAACCAGTCTTACCAAGGGGTATCGGGTTGCCCAGGT-AACTGGGTTGAGGAGGTCAGATA
HaSE3.9   168 TGGTAGTCGTTACGGGTAGTCAGAAGCCAGAAAGTCTGACAACCAGTCTTACCAAGGGGTATCGGGCTGCCCAGGT-AACTGGGTTGAGGAGGTCAGATA
HaSE3.10  168 TGGTAGTCGTTACGGGTAGCCAGAAGCCAGAAAGTCTGACAACCAGTCTTACCAAGGGGTATCGGGTTGCCCAGGT-AACTAGGTTGAGGAGGTCAGATA
HaSE3.11  158 TGGCAGTCGTTACGGGTAGTCAGAAGCCAGATAGTCTGACAACCAGTCTTAACAATGGATGTCGAGTTGCCTGAGTAACCTGGGTTGAGGAGCTCAGATA
HaSE3.12  143 TGGCAGTCGTTACGGATAGTCAGAAGCCAGTAAGCCTGACAACCAGTCTAACCAAAGGGTATTGAGTTGCCCGGGT-AAATGGGTTGAGGAGTTCAGATA
HaSE3.13  162 TAGCAGTCGTTACC--TAGTCAGAAGCCAATCAGCCCGACGT---GTCTTATCAAGGG--------TTGCCCAAGTTAACTGGGTTGAGGAGGTCAGATA
HaSE3.14  146 TAGCAGTCGTTACGGGTAGTCAGTAGCCAGTAAGTCGGACAACCAGTCTTACCTAGGGGTATCGGGTTGCCCGGAT-AACTCAGTTGAGAAGGTCAGATA
HaSE3.15  169 TGGCAGTCATTACGGGTAGTCAGAAGCCAAAA-----GATTACCAGTCATACCAAGGG---------------GGT-AACCAGATCGAAAAGGTCAGACA
HaSE3.16  167 TGGCAGTCGTTACGTGTAGTCAGAAGACAGAAGGGCTGTTAAATCGTCTCAATAACGG--------TTGCCCGGGT-AACTGTGTTAAGCAGGTTACATA


HaSE3CS   267 GG----------CAGTCGCTCCTTGTAAAACACTGGTACTCAGCTGCATCCGGTT--AGACTGGAAGCCGACCCCAAAATAGTTTGGAAAAGGC------
HaSE3.1   289 GAA----------AACTGCTCCATGTGGTACACTGGTACT-----ACATA-GGTTTAAGATTGGTAGCCGACCTCAACATAGTTTGGAAAAGGCAAAACA
HaSE3.2   267 GG----------CAGTCGCTCCTTGTAAAACCCTGGTACTCAGCTGCATCCGGTC--AGACTGGAAGCTGACCCCAAAATAGTTTGGAAAAGGC------
HaSE3.3   267 GG----------CAGTCGCTCCTTGTAAAACATTGGTACTCAGCTGCATCCGGTT--AGACTGGAAGCCGACCCCAAAATAGTTTGGAAAAGGC------
HaSE3.4   267 GG----------CAGTCGCTCCTTGTAAAACCCTGGTACTCAGCTGCATCCGGTT--AGACTGGAAGCCGACCCCAACATAGTTTGGAAAAGGC------
HaSE3.5   267 GA----------CAGTCGCTCCTTGTAAAACCCTGGTACTCAGCTGCATCCGGTT--AGACTGGAAGCCGACCCCAAAATAGTTTGGAAAAGGC------
HaSE3.6   267 GG----------CAGTCGCTCCTTGTAAAACCCTGGTACTCAGCTGCATCCGGTT--AGACTGGAAGCCGACCCCAAAATAGTTTGGAAAAGGC------
HaSE3.7   267 GG----------CAGTCGCTCCTTGTAAAACCCTGGAACTCAGCTGCATCCGGTT--AGACTGGAAGCCGACCCCAAAATAGTTTGGAAAAGGC------
HaSE3.8   267 GG----------CAGTCGCTCCTTGTAAAGCACTGGTACTCAGCTGCATCCGGTT--AGACTGGAAGCCGACCCCAAAATAGTTTGGAAAAGGC------
HaSE3.9   267 GG----------CAGTCGCTCCTTGTAAAACATTGGTACTCAGCTGCATCCGGTT--AGACTGGAAGCCGACCCCAAAATAGTTTGGAAAAGGC------
HaSE3.10  267 GG----------CAGTCGCTCCTTGTAAAGCACTGGTACTCT-CTACATCCGGTT--AGACTGGAAGCCGACCCCAAAATAGTTGGGAAAAGGC------
HaSE3.11  258 GG----------CAGTCGCTTCCTGTAAAACACTGGTAACCAGCTGCATCCGGTT--AGACTGGAAGC---CCCCAAAATAGTTTGGAAAAGGC------
HaSE3.12  242 GATATAGATAGGCAGTCGCCCCTTGCGGCACACTGGTACTCAGCTGCATGCTGTT--AGACTGGAAGCCAACCCCAAAATAGTTTGGAAAAGGC------
HaSE3.13  249 GGG---------CAGTCGCTCCATGTAAAACACTGATACTCAGCTGCATCCAGTT--AGACTGGAAGTCGACTCCAAAATAGTTTGGAAAAGGC------
HaSE3.14  245 GCTCTA-----------------TGTGGCACACTGGTACTCAGCTGCATCCGATT--AGACTGGAAGCCGACCCCAAAATAGTTTGGAAAAGGC------
HaSE3.15  248 GG----------------CTTAGTGTGAAACACTGTTACTCAG---------ATT--AGGCTGGAAGCCGACATCAAAATAGTTTGGAAAAGGC------
HaSE3.16  258 GAA----------AACTGCTCCATGTGGTACACTGGTACT-----ACATA-GGTT-AAGATTGGTAGCCGACCTCAAAATAGTTTGGAAAAGGC------


HaSE3CS       ---------------------------
HaSE3.1   373 GCTGGTCAAAGGCCGGATCATCTCCTT
HaSE3.2       ---------------------------
HaSE3.3       ---------------------------
HaSE3.4       ---------------------------
HaSE3.5       ---------------------------
HaSE3.6       ---------------------------
HaSE3.7       ---------------------------
HaSE3.8       ---------------------------
HaSE3.9       ---------------------------
HaSE3.10      ---------------------------
HaSE3.11      ---------------------------
HaSE3.12      ---------------------------
HaSE3.13      ---------------------------
HaSE3.14      ---------------------------
HaSE3.15      ---------------------------
HaSE3.16      ---------------------------

Figure S5. Alignments of the identified HaSE3 sequences in Helicoverpa armigera. The sequence on the top line is the consensus sequence of the HaSE3 family. Nucleotides shaded in black are conserved across sequences.
