# Supplementary material for: Characterization of Three Novel SINE Families with Unusual Features in Helicoverpa armigera
Source: PLoS One. 2012 Feb 3;7(2):e31355. doi: 10.1371/journal.pone.0031355 (PMC3272025; doi:10.1371/journal.pone.0031355)
Supplement: Table S1 — Primers used for genome walking and PCR amplification of HaSE3 elements. (RTF) [file pone.0031355.s006.rtf]

Table S1. Primers used for genome walking and PCR amplification of HaSE3 elements.
Primer name	Sequence (5' to 3')	Sequence information	
177.6ae12SPF1	CTACATGCCGTTTGGTGAAGGTCC	First outer primer for genome walking	
178.6ae12SPF2	TGCAAATGGCGGCAGGTCTGTTAA	Second nested primer for genome walking	
179.6ae12SPF3	AGAACTGGTCTTCGAGCCAGGAG	Third nested primer for genome walking	
527.SINE3F1	AGAAGAGCTCKTGRCYAAGT	Forward primer for HaSE3 PCR	
528.SINE3R1	GCCTTTTCCMAACTATKTTG	Reverse primer for HaSE3 PCR	
