# Supplementary material for: Characterization of Three Novel SINE Families with Unusual Features in Helicoverpa armigera
Source: PLoS One. 2012 Feb 3;7(2):e31355. doi: 10.1371/journal.pone.0031355 (PMC3272025; doi:10.1371/journal.pone.0031355)
Supplement: Table S2 — Genes found in the flanking sequences of full length HaSE1 and HaSE2. (RTF) [file pone.0031355.s007.rtf]

Table S2. Genes found in the flanking sequences of full length HaSE1 and HaSE2.
Name 	5'-Flanking sequence	3'-Flanking sequence	Insertion region	
	Description (Gene function/ organism)	GenBank
Accession No.	E-value	Description (Gene function/ organism)	GenBank
Accession No.	E-value		
HaSE1.1	No significant hit (only 500 bp available)			cytochrome P450 CYP6AE12/ Harpegnathos saltator,	ABB79054	0	Intergenic	
HaSE1.2-1	Aminopeptidase N/ Harpegnathos saltator	EFN87052	2e-29	Aminopeptidase N/ Harpegnathos saltator	EFN87052	4e-08	Intronic	
HaSE1.2-2	ABC transporter family C protein ABCC3/ Heliothis subflexa	ADH16743	0.002	Aminopeptidase N/ Harpegnathos saltator	EFN87052	4e-08	Intronic	
HaSE1.3-1	Transcription factor glial cells missing/ Harpegnathos saltator	EFN87857	5e-61	endonuclease-reverse transcriptase/ Heliconius        melpomene	CBA11992	4e-41	Intergenic*	
HaSE1.3-2	reverse transcriptase/ Aedes aegypti	AAZ15237	0.005	No significant hit			Intergenic*	
HaSE1.4	endonuclease-reverse transcriptase/ Bombyx mori	ADI61810	0	transcription factor Ets/ Bombyx mori	NP_001036902	1e-108	Intergenic*	
HaSE1.5-1	midgut aminopeptidase APN1/ Helicoverpa armigera	AAN75693	1e-122	midgut aminopeptidase APN1/ Helicoverpa armigera	AAN75693	1e-81	Intronic	
HaSE1.5-2	midgut aminopeptidase APN1/ Helicoverpa armigera	AAN75693	1e-122	midgut aminopeptidase APN1/ Helicoverpa armigera	AAN75693	6e-81	Intronic	
HaSE1.5-3	midgut aminopeptidase APN1/ Helicoverpa armigera	AAN75693	1e-122	midgut aminopeptidase APN1/ Helicoverpa armigera	AAN75693	6e-81	Intronic	
HaSE1.5-4	aminopeptidase N1/ Helicoverpa armigera 	AAQ57406	8e-88	aminopeptidase N1/ Helicoverpa armigera	AAQ57406	3e-82	Intronic	
HaSE1.6	Glutaminase kidney isoform, mitochondrial/ Harpegnathos saltator	EFN75602	9e-28	Glutaminase kidney isoform/ Harpegnathos saltator	EFN75602	5e-59	Intronic	
HaSE1.7	39S ribosomal protein L51, mitochondrial/ Culex quinquefasciatus	EDS35014	5e-16	zinc finger protein 347-like/ Bos taurus	DAA19471	6e-24	Intergenic	
HaSE1.8	fat body aminopeptidase/ Spodoptera litura	ABN04204	1e-44	fat body aminopeptidase/ Spodoptera litura	ABN04204	5e-18	Intronic	
HaSE1.9	aminopeptidase N-7/ Helicoverpa armigera	ACA35024	2e-61	aminopeptidase N-7/ Helicoverpa armigera	ACA35024	3e-55	Intronic	
HaSE1.10-1	aminopeptidase N1/ Helicoverpa armigera	AAQ57406	8e-88	aminopeptidase N4/ Helicoverpa armigera	AAO23562 	0	Intergenic	
HaSE1.10-2	aminopeptidase N/ Helicoverpa armigera	AAN04899	2e-84	midgut aminopeptidase APN1/ Helicoverpa armigera	AAN75693	6e-72	Intergenic	
HaSE1.10-3	aminopeptidase N/ Helicoverpa armigera	AAN04899	2e-84	midgut aminopeptidase APN1/ Helicoverpa armigera	AAN75693	6e-72	Intergenic	
HaSE1.10-4	aminopeptidase N/ Helicoverpa armigera	AAN04899	2e-84	midgut aminopeptidase APN1/ Helicoverpa armigera	AAN75693	6e-72	Intergenic	
HaSE1.11-1	aminopeptidase N1/ Helicoverpa armigera	ACA35024 	2e-61	aminopeptidase N1/ Helicoverpa armigera	ACA35024	6e-24	Intronic	
HaSE1.11-2	aminopeptidase N-7/ Helicoverpa armigera	ACA35024	2e-61	aminopeptidase N-7/ Helicoverpa armigera	ACA35024	6e-22	Intronic	
HaSE1.11-3	aminopeptidase N-7/ Helicoverpa armigera	ACA35024	2e-61	aminopeptidase N-7/ Helicoverpa armigera	ACA35024	6e-22	Intronic	
HaSE1.11-4	aminopeptidase N-7/ Helicoverpa armigera	ACA35024	2e-61	aminopeptidase N-7/ Helicoverpa armigera	ACA35024	6e-22	Intronic	
HaSE1.12	similar to Trim9 CG31721-PA/ Tribolium castaneum	XP_974556	0.006	imilar to Trim9 CG31721-PA/ Tribolium castaneum	XP_974556	7e-68	Intronic	
HaSE1.13
	tigger transposable element-derived protein 4-like
/Acyrthosiphon pisum	XP_001946731            	9e-13
	KRAB-A domain-containing protein 2-like /Acyrthosiphon pisum	XP_003242818	1e-15
	Intergenic*	
HaSE1.14 	similar to helentron 5 helitron-like transposon  replicase/helicase/endonuclease/ Hydra magnipapillata	XP_002169581	0.087	endonuclease-reverse transcriptase/ Bombyx mori	ADI61817
	0.025	Intergenic*	
HaSE1.15	hypothetical protein TcasGA2_TC014264/ Tribolium castaneum	EFA04035	0.002
	hypothetical protein TcasGA2_TC014264/ Tribolium castaneum	EFA04035
	1e-10	Intronic	
HaSE1.16	No significant hit (only 5596 bp available)			hypothetical protein TcasGA2_TC014264/ Tribolium castaneum	EFA04035
	3e-13
	Intergenic	
HaSE1.17	similar to Trim9 CG31721-PA/ Tribolium castaneum	XP_974556	7e-68	similar to Trim9 CG31721-PA / Tribolium castaneum	XP_974556	0.006	Intronic	
HaSE1.18-1	antennal cytochrome P450 CYP9/ Mamestra brassicae	AAR26518	6e-62	cytochrome P450/ Helicoverpa armigera	ABB69055	1e-147	Intergenic	
HaSE1.18-2	No significant hit			No significant hit			Intergenic	
HaSE1.19	No significant hit			endonuclease-reverse transcriptase/ Bombyx mori	ADI61822	0.0	Intergenic*	
HaSE1.20	glucose-6-phosphate isomerase/ Spodoptera exigua	ACV97159	4e-26	flap endonuclease-1/ Bombyx mori	ACY92094	2e-22	Intergenic	
HaSE1.21	endonuclease-reverse transcriptase/ Bombyx mori	ADI61822	4e-134	endonuclease-reverse transcriptase/ Bombyx mori	ADI61811	6e-09	Intergenic*	
HaSE1.22	Microsatellite locus	Intergenic	
HaSE2.1	Intron of cadherin-like protein1	Intronic	
HaSE2.2-1	aminopeptidase N-7/ Helicoverpa armigera	ACA35024	3e-64	aminopeptidase N-7/ Helicoverpa armigera 8617 bp	ACA35024	1e-49	Intronic	
HaSE2.2-2	aminopeptidase N-7/ Helicoverpa armigera	ACA35024	3e-64	aminopeptidase N-7/ Helicoverpa armigera	ACA35024	2e-49	Intronic	
HaSE2.3	No significant hit			parathyroid hormone-responsive B1/ Helicoverpa armigera	AEH16631	1e-148	Intergenic	
HaSE2.4	endonuclease-reverse transcriptase/ Bombyx mori	ADI61812	6e-88	sodium channel alpha subunit/ Helicoverpa armigera	ABE60888	1e-08	Intergenic*	
HaSE2.5	Protein FAM50-like protein/ Harpegnathos saltator	EFN87424	7e-29	endonuclease-reverse transcriptase/ Bombyx mori	ADI61830	2e-73	Intergenic*	
HaSE2.6	Pre-mRNA-processing factor 40-like protein B/ Acromyrmex echinatior	EGI60246	1e-22	Pre-mRNA-processing factor 40-like protein B/ Acromyrmex echinatior	EGI60246	5e-49
	Intronic	
HaSE2.7	No significant hit (only 1207 bp available)			similar to heparan-alpha-glucosaminide N-acetyltransferase/ Tribolium castaneum	XP_974454
	8e-17	Intergenic	

* Transposable elements were found in flanking sequences on one side or both sides.
