# Supplementary material for: Characterization of Three Novel SINE Families with Unusual Features in Helicoverpa armigera
Source: PLoS One. 2012 Feb 3;7(2):e31355. doi: 10.1371/journal.pone.0031355 (PMC3272025; doi:10.1371/journal.pone.0031355)
Supplement: Table S3 — Genes found in the flanking sequences of 5′-truncated HaSE1. (RTF) [file pone.0031355.s008.rtf]

Table S3. Genes found in the flanking sequences of 5′-truncated HaSE1.
Name 	5'-Flanking sequence	3'-Flanking sequence	Insertion region	
	Description (Gene function/ organism)	GenBank
Accession No.	E-value	Description (Gene function/ organism)	GenBank
Accession No.	E-value		
HaSE1.23	fructose-biphosphate aldolase/ Glossina morsitans morsitans 	ADD19731 	 3e-04 	reverse transcriptase/ Ostrinia nubilalis 	ABO45233	3e-09 	Intergenic*	
HaSE1.24	reverse transcriptase/ Ostrinia nubilalis 	ABO45233	3e-09	fructose-biphosphate aldolase/ Glossina morsitans morsitans	ADD19731	1e-04 	Intergenic*	
HaSE1.25	No significant hit			No significant hit			Intergenic	
HaSE1.26	similar to reverse transcriptase-like protein/ Hydra magnipapillata	XP_002167955	3e-11	No significant hit			Intergenic*	
HaSE1.27 	No significant hit			hypothetical protein TcasGA2_TC014264/Tribolium castaneum	EFA04035	2e-11 	Intergenic	
HaSE1.28	No significant hit			Aminopeptidase N/ Harpegnathos saltator	EFN87052 	 1e-17	Intergenic	
HaSE1.29-1	Aminopeptidase N/ Harpegnathos saltator	EFN87052 	3e-07	No significant hit (only 490 bp available)			Intronic	
HaSE1.29-2	Aminopeptidase N/ Harpegnathos saltator	EFN87052	3e-07	Aminopeptidase N/ Harpegnathos saltator	EFN87052	3e-15	Intronic	
HaSE1.30	No significant hit			Aminopeptidase N/ Harpegnathos saltator	EFN87052	2e-24	Intergenic	
HaSE1.31 	hypothetical protein TcasGA2_TC009470/ Tribolium castaneum	EFA06559	2.8	hypothetical protein TcasGA2_TC009470/ Tribolium castaneum	EFA06559	1e-25	Intronic	
HaSE1.32	glucose-6-phosphate isomerase/ Spodoptera exigua	ACV97159	 4e-15	glucose-6-phosphate isomerase/ Spodoptera exigua	ACV97159	9e-22	Intronic	
HaSE1.33	dmX-like protein 1-like/ Bombus terrestris	XP_003402901	3e-16 	dmX-like protein 1-like/ Bombus terrestris	XP_003402901	3e-12	Intronic	
HaSE1.34	No significant hit  (only 9638 bp available)			aminopeptidase N/ Culex quinquefasciatus	EDS36843	1e-35	Intergenic	
HaSE1.35	PREDICTED: GMP reductase 2-like isoform 1/ Apis mellifera	XP_624694	1e-07 	endonuclease-reverse transcriptase HmRTE-e01 / Heliconius melpomene	CBA11992	1e-165	Intergenic*	
HaSE1.36	PREDICTED: GMP reductase 2-like isoform 1/ Apis mellifera	XP_624694	3e-17 	reverse transcriptase/ Anopheles gambiae	BAC82595	2e-161 	Intergenic*	
HaSE1.37 	Enterin neuropeptide/ Acromyrmex echinatior	EGI62941	2e-05	No significant hit			Intergenic	
HaSE1.38	glucose-6-phosphate isomerase/ Spodoptera exigua	ACV97159	2e-23	PREDICTED: similar to heparan-alpha-glucosaminide N-acetyltransferase/ Tribolium castaneum	XP_974454	2e-16	Intergenic	
HaSE1.39	GMP reductase 1/ Harpegnathos saltator	EFN87499	9e-30	GMP reductase 1/ Harpegnathos saltator	EFN87499	3e-09	Intronic	
HaSE1.40	reverse transcriptase/ Ostrinia nubilalis	ABO45239	2e-08	PREDICTED: similar to reverse transcriptase-like protein/ Hydra magnipapillata	XP_002167955	2e-10	Intergenic*	
HaSE1.41	reverse transcriptase/ Bombyx mori	BAC57926	4e-19	cytochrome P450 CYP4L4/ Mamestra brassicae	AAL48300	5e-08	Intergenic*	
HaSE1.42	cysteine-rich/pacifastin venom protein 2/ Nasonia vitripennis	NP_001154996	4e-9	cysteine-rich/pacifastin venom protein 2/ Nasonia vitripennis	NP_001154996	0.003	Intronic	
HaSE1.43	conserved hypothetical protein/ Aedes aegypti	EAT42315	0	MSL2 protein/ Bombyx mori	ABQ51915	5e-176	Intergenic	

* Transposable elements were found in flanking sequences on one side or both sides.
